# Molecular subtype identification and prognosis stratification by a metabolism-related gene expression signature in colorectal cancer

**DOI:** 10.1186/s12967-021-02952-w

**Published:** 2021-06-30

**Authors:** Dagui Lin, Wenhua Fan, Rongxin Zhang, Enen Zhao, Pansong Li, Wenhao Zhou, Jianhong Peng, Liren Li

**Affiliations:** 1 Department of Colorectal Surgery, State Key Laboratory of Oncology in South China, Collaborative Innovation Center of Cancer Medicine, Sun Yat-Sen University Cancer Center, 651 Dongfeng Road East, Guangzhou, 510060 Guangdong People’s Republic of China; 2 Geneplus-Beijing, Beijing, 102206 China

**Keywords:** Colorectal cancer, Metabolic risk signature, Nomogram, Metabolism-related subtypes

## Abstract

**Background:**

Metabolic reprograming have been associated with cancer occurrence and progression within the tumor immune microenvironment. However, the prognostic potential of metabolism-related genes in colorectal cancer (CRC) has not been comprehensively studied. Here, we investigated metabolic transcript-related CRC subtypes and relevant immune landscapes, and developed a metabolic risk score (MRS) for survival prediction.

**Methods:**

Metabolism-related genes were collected from the Molecular Signatures Database and metabolic subtypes were identified using an unsupervised clustering algorithm based on the expression profiles of survival-related metabolic genes in GSE39582. The ssGSEA and ESTIMATE methods were applied to estimate the immune infiltration among subtypes. The MRS model was developed using LASSO Cox regression in the GSE39582 dataset and independently validated in the TCGA CRC and GSE17537 datasets.

**Results:**

We identified two metabolism-related subtypes (cluster-A and cluster-B) of CRC based on the expression profiles of 539 survival-related metabolic genes with distinct immune profiles and notably different prognoses. The cluster-B subtype had a shorter OS and RFS than the cluster-A subtype. Eighteen metabolism-related genes that were mostly involved in lipid metabolism pathways were used to build the MRS in GSE39582. Patients with higher MRS had worse prognosis than those with lower MRS (HR 3.45, *P* < 0.001). The prognostic role of MRS was validated in the TCGA CRC (HR 2.12, *P* = 0.00017) and GSE17537 datasets (HR 2.67, *P* = 0.039). Time-dependent receiver operating characteristic curve and stratified analyses revealed the robust predictive ability of the MRS in each dataset. Multivariate Cox regression analysis indicted that the MRS could predict OS independent of TNM stage and age.

**Conclusions:**

Our study provides novel insight into metabolic heterogeneity and its relationship with immune landscape in CRC. The MRS was identified as a robust prognostic marker and may facilitate individualized therapy for CRC patients.

**Supplementary Information:**

The online version contains supplementary material available at 10.1186/s12967-021-02952-w.

## Background

Colorectal cancer (CRC) is one of the most common malignancies, ranking as the second leading cause of cancer-related death worldwide [1]. In the past few decades, the occurrence and mortality of CRC have decreased steadily due to advanced screening programs comprising fecal occult blood testing, direct colonic visualization, and noninvasive imaging techniques [1, 2]. However, the 5-year survival rate of CRC remains dismal [1, 3]. Currently, the tumor–node–metastasis (TNM) system is widely used for risk assessment and therapy decision making in clinical settings [4]. However, the relapse and death risks may vary vastly even in patients with the same clinicopathological features due to the high level of molecular heterogeneity [4, 5]. Hence, novel prognostic factors to identify CRC patients’ risk more accurately are urgently needed.

Cellular metabolism is an essential biochemical process used to meet the basic need for cell survival and proliferation. Emerging studies suggest that metabolic reprogramming promotes tumor growth and progression [6–8]. On the one hand, cancer cells can outcompete adjacent normal cells for nutrients, including glucose, amino acids, and glutamine, to maintain high rates of cell division [9]. On the other hand, cancer cells can produce immunosuppressive metabolites through cross-talk with stromal and immune cells within the tumor microenvironment, thereby inducing immune dysfunction and tumor progression [6, 10, 11]. In addition to findings on metabolomics, which is defined as the comprehensive analysis of all small molecule metabolites in a biological system [12], recently, increasing number of studies have recently focused on the relationship between metabolism and survival at the transcriptional level. For example, both Peng et al. and Sinkala et al. conducted pan-cancer research to characterize tumor subtypes based on the expression patterns of metabolic pathway-related genes and showed distinct survival among the subtypes [13, 14]. Moreover, metabolic gene set-based prognostic signatures have been proposed in head and neck carcinoma [15, 16] and neuroblastoma [17]. However, studies focusing on subtype characterization and risk signatures based on metabolism-related genes in CRC remain limited.

In this work, we used the GSE39582 dataset to classify CRC patients based on metabolism-related gene expression profiles. Then, we compared the prognosis and immune landscape and elucidated the underlying pathway enrichment between subtypes. Furthermore, we attempted to establish a metabolic risk score (MRS) in the GSE39582 dataset and validated it in the GSE17537 and The Cancer Genome Atlas (TCGA) CRC datasets. The associations of clinical immune features with MRS were further explored. Consequently, a prognostic nomogram integrating the MRS and clinical risk factors was developed and showed decent predictive performance in estimating mortality risk.

## Methods

### Data sources and preprocessing

We downloaded the raw CEL files of the GSE39582 [18] and GSE17537 datasets from Gene Expression Omnibus (GEO) database (https://www.ncbi.nlm.nih.gov/geo/) and normalized using the robust multiarray averaging method by the “affy” R package [19]. Moreover, we retrieved the RNA sequencing (RNA-seq) profiles and clinical information of CRC patients from the TCGA database (https://portal.gdc.cancer.gov/) and transformed the raw count data to transcripts per kilobase million (TPM) values to make the samples more comparable [20]. To obtain reliable conclusions from the downstream analysis, we excluded samples with a follow-up time less than 3 months in GSE39582 and TCGA datasets, and eventually included 1142 patients in the study, of which 540, 548, and 54 patients were from the GSE39582, TCGA CRC and GSE17537 datasets, respectively (Additional file 1: Table S1; Additional file 2: Table S2; Additional file 3: Table S3). The TNM stages for GSE39582 and TCGA datasets were redefined according to the America Joint Committee on Cancer (AJCC) 8th classification system and the following criteria: first, samples with any uncertain pathological T, N, and M stages were classified as samples with unknown stages; second, samples that showed clear T, N, and M stages separately but lacked an available summarized stage were reclassified.

### Identification of metabolism-related subtypes

A comprehensive list of 2923 metabolism-related genes involved in 117 metabolic pathways were retrieved from the Molecular Signatures Database (MSigDB) and were used to screen out survival-related metabolic genes with log-rank *P*-value < 0.05. Further, the expression profiles of 539 survival-related metabolic genes were employed to performed k-means unsupervised clustering by the “ConsensusClusterPlus” package with 1000 repetitions [21, 22].

### Estimation of immune infiltration and immune-related pathway activity

To quantify the immune infiltration for each sample, single sample gene set enrichment analysis (ssGSEA) was applied via the “GSVA” package based on 28 immune cell gene sets and 17 immune-related pathways that retrieved from previous study [23, 24] (Additional file 4: Table S4; Additional file 5: Table S5). Additionally, ESTIMATE algorithm was used to compute the stromal score, immune score, ESTIMATE score, and tumor purity for each tumor sample [25].

### Differentially expressed gene (DEG) and pathway enrichment analyses

The gene expression divergence between subtypes was explored using the “limma” R package. The genes with fold change > 2 and Benjamini–Hochberg-adjusted *P* < 0.05 were considered DEGs. We also conducted Kyoto Encyclopedia of Genes and Genomes (KEGG) and Gene Ontology (GO) enrichment analyses using the “clusterProfiler” package to elucidate underlying subtype-related functional pathways. Moreover, the C2 gene set (“c2.cp.kegg. v7.1. symbols”) was used to perform gene set enrichment analysis (GSEA) between subtypes through Java GSEA software with random sample permutations of 1000 and the results were visualized by the “enrichplot” R package.

### Construction and validation of the MRS

To construct the MRS, we first applied univariate Cox regression analysis to detect the survival-related genes out of 2923 metabolism-related genes with log-rank *P* < 0.01. Then, the survival-related metabolic genes were further narrowed down by the least absolute shrinkage and selection operator (LASSO) penalty method, with optimal parameter λ tuned by ten-fold cross-validation. The candidate genes were divided into high or low expression groups based on the optimal cutoff values calculated by the “survminer” package, and their prognostic values were confirmed by survival analyses. Then, the MRS was calculated for each patient using the following formula:

$${\text{MRS}} = \sum {{\text{LASSO}}\;{\text{coefficient}}\left( {{\text{gene}}} \right)*{\text{expression}}\;{\text{value}}\left( {{\text{gene}}} \right)}$$

where expression value represents the normalized value of the selected genes that were normalized by log 2 and z-score transformations. The coefficients of the selected genes, their involved metabolic pathways, and the details of the formula are presented in Additional file 7: Table S7. We divided the patients in each dataset into high- and low-MRS groups based on the cohort-specific median value and compared the difference in survival rates via the Kaplan–Meier survival curve. A time-dependent receiver operating characteristic (ROC) curve was drawn to evaluate the sensitivity and specificity of the MRS in each dataset.

### Correlations of the MRS with clinical characteristics and immune infiltration

The differences in the MRS in term of various clinical parameters were compared via the Mann–Whitney U test for two groups or the Kruskal–Wallis test for more than two groups. We also estimated the differences in the infiltration levels of 28 immune cells between the high- and low-MRS groups via the same method. The associations of the MRS with key immune cell types such as activated CD4 T cells, activated CD8 T cells, myeloid-derived suppressor cells (MDSCs), immature dendritic cells, and T follicular helper cells were further confirmed by the Spearman correlation test.

### Establishment and validation of a prognostic nomogram

Univariate and multivariate Cox regression analyses were applied to evaluate the independent prognostic value of the MRS. Then, a prognostic nomogram was constructed by integrating the MRS and the clinical factors identified by multivariate regression analysis. Calibration curves for 3-year and 5-year survival were generated to evaluate the deviation between the nomogram and the ideal model. ROC curves were generated and the areas under the curves (AUCs) were computed to assess the predictive capacity of the nomogram integrating age, TNM stage, and the established MRS. The prognostic value of the nomogram was also compared with that of the MRS as a continuous variable by the concordance index (C-index) and presented by the restricted mean survival (RMS) curve [26]. RMS represents the life expectancy at 10 years (120 months) of patients with different risk scores. The RMS time ratio between the low- and high-risk groups was computed for the nomogram and MRS separately [27]. A higher RMS time ratio represents a larger prognostic difference. A decision-curve analysis (DCA) plot was used to measure the standardized net benefit of the nomogram.

### Statistical analysis

All statistical analyses were performed by R software (version 3.62). The primary endpoint analyzed in this study was overall survival (OS), which defined as the interval between the date of diagnosis and the date of death, and the secondary endpoint was relapse-free survival (RFS), which was defined as the interval between the date of surgery and the date of the first relapse. The individualized consensus molecular subtype (CMS) for each sample was assessed by the “CMScaller” package [28] with a default false discovery rate (FDR) of 0.05 (Additional file 8: Figure S1). ROC curves were generated with the “survivalROC” package. C-indexes were calculated with the “survcomp” package and compared with “compareC” package [26]. The RMS curve and RMS time ratio were estimated with the “survival” and “survRM2” packages [27]. The nomogram was developed with the “rms” package. All statistical tests were two-sided, and a *P*-value < 0.05 was considered significant unless otherwise specified.

## Results

### Patient characteristics

We included 1142 patients from the GSE39582, GSE17537 and TCGA CRC datasets. Of the 540 patients in the GSE39582 dataset, 32 (5.92%), 241(44.63%), 189 (35.00%), and 56 (10.37%) were in stage I, II, III, and IV respectively. Moreover, 233 (43.15%) patients had undergone adjuvant chemotherapy and 68 (12.59%) patients were diagnosed as dMMR. The proportion of TP53 mutated, KRAS mutated and BRAF mutated patients was 34.7%, 38.15% and 8.33% respectively. The median follow-up time was 53 (interquartile range [IQR], 30–81) months. Of the 548 patients from the TCGA dataset, 83 (15.15%), 187 (34.12%), 134 (24.45%), and 72 (13.14%) were in stage I, II, III, and IV respectively. Additionally, 37 (6.75%) patients had received radiotherapy and 70 (12.77%) were diagnosed as MSI-H. The median follow-up was 24.18 (IQR, 14.41–36.92) months. Of the patients from the GSE17537 dataset, 4 (7.41%), 15 (27.78%), 19 (35.18%) and 16 (29.63%) were in stage I, II, III, and IV respectively. Most patients (46.30%) were diagnosed with moderately differentiated carcinoma and the median follow-up was 51.16 (IQR, 32.66–60.03) months. The details of the patient’s characteristics were summarized in Additional file 1: Tables S1; Additional file 2: Table S2; Additional file 3: Table S3, respectively.

### Molecular subtypes based on metabolism-related genes and prognostic differences

**Fig. 1:**
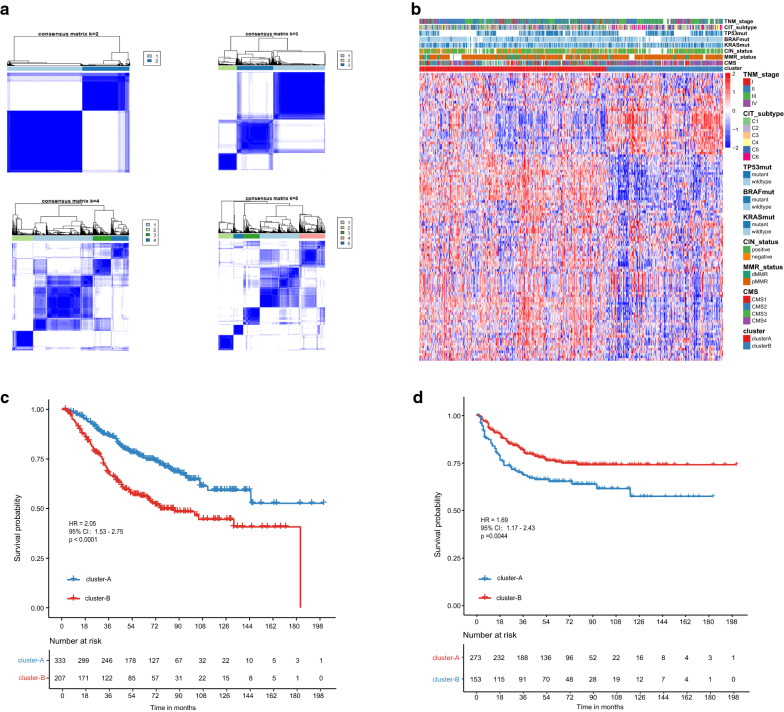
Metabolism-related subtype construction and their prognostic value in the GSE39582 dataset. **a** Consensus matrix heatmap to cluster patients into 2 to 5 clusters, showing the clustering stability after the 1000 times k-means cluster approach. **b** Heatmap of metabolic pathway expression patterns of two clusters. **c**, **d** Kaplan–Meier curves of overall survival (**c**) and relapse-free survival (**d**) between the two clusters. RFS survival analysis was conducted in stage II/III patients. *HR* hazard ratio, *CI* confidence interval; *P* value was measured by the log-rank test

We collected 117 metabolism-related pathways comprising 2923 genes from MSigDB. Using an unsupervised consensus algorithm, we identified two robust subtypes (333 patients in cluster-A and 207 patients in cluster-B) in GSE39582 based on 539 metabolism-related genes (log-rank *P*-value < 0.05; Additional file 6: Table S6) first determined by univariable Cox regression analysis (Fig. 1a). In addition, we quantified the expression profiles of the 117 metabolism-related pathways via ssGSEA and found that patients in cluster-B were more likely to have C4C6 subtype and CMS4 subtype (Fig. 1b). Survival analysis indicated that cluster-B was associated with worse OS than cluster-A (hazard ratio [HR] 2.05, 95% confidence interval [CI], 1.53–2.75; *P* < 0.0001; Fig. 1c). A similar risk of RFS was observed in stage II and III patients (HR 1.69, 95% CI 1.17–2.43; *P* = 0.0044; Fig. 1d).

### Immune profiles of the subtypes

**Fig. 2:**
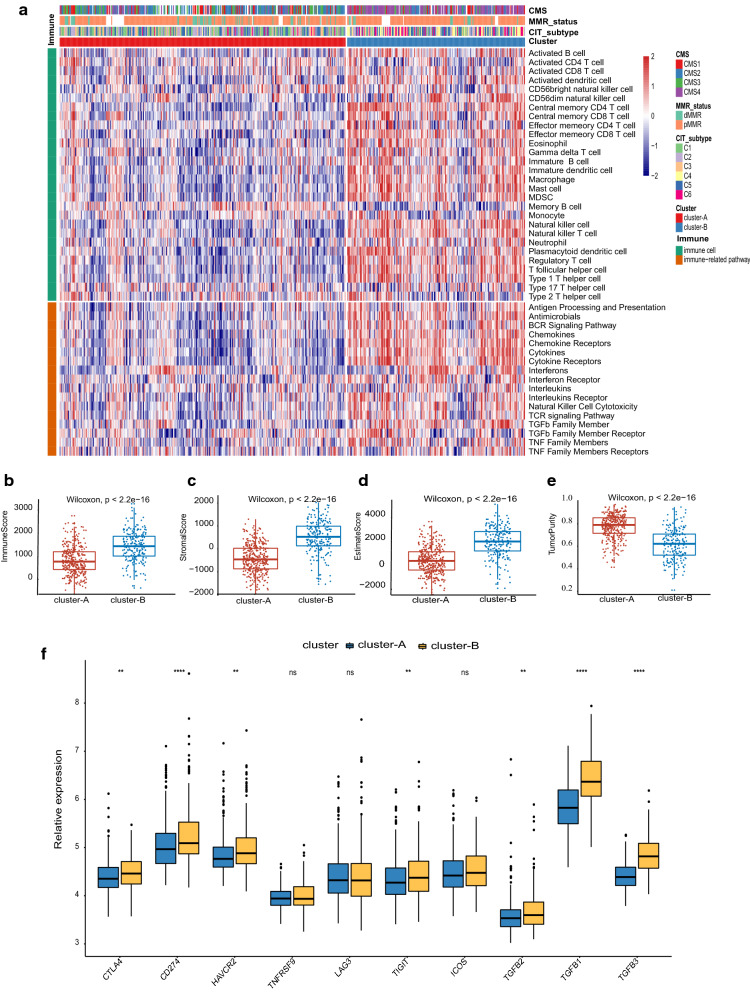
The different immune profiles between the two molecular subtypes. **a** Discrepancies of immune cell infiltration and expression of immune-related pathways between cluster-A and cluster-B. **b**–**e** Distributions of immune score, stromal score, EATIMATE score, and tumor purity between cluster-A and cluster-B. The distance of both ends of boxes represents the interquartile range of values and the thick line represents the median value. The significance of the Mann–Whitney test is shown by an asterisk (*P < 0.05; **P < 0.01; ***P < 0.001). **f** Expression variation in gene signatures for dysfunctional T cell states and TGF beta-encoding genes between subtypes

Based on the gene sets of 28 tumor-infiltrating cells and 17 classical immune-related pathways collected from published studies [23, 24], the ssGSEA results showed that cluster-B was markedly richer not only in innate immune cell infiltration including mast cells, macrophages, and MDSCs, but also, in adaptive immune cell infiltration, such as activated B cells, activated CD8 T cells, activated dendritic cells effector memory CD4 T cells, and effector memory CD8 T cells than cluster-A (Fig. 2a). Only activated CD4 T cells and memory B cells significantly increased in cluster-A when compared with cluster-B. Intriguingly, cluster-B also showed a higher abundance of immune-related pathways than cluster-A (Fig. 2a). Additionally, cluster-B was associated with an evaluated immune score, stromal score, EASTIMATE score, and decreased tumor purity while cluster-A showed the opposite trends (Fig. 2b–e), which confirmed the above findings (Fig. 2a). Given that the T cell dysfunction state and immunosuppressive phenotype are characterized by high expression levels of immune checkpoint-relevant transcripts, such as CTLA-4, PDL1 (CD274), HAVCR2 (TIM-3), TNFRSF9 (4-1BB or CD137), LAG3, TAGIT and ICOS, and TGF beta encoding genes including TGFB1, TGFB2, and TGFB3 [29, 30], we speculated that the worse prognosis of cluster-B was driven by immunosuppressive signals. The higher expression of CTLA-4, CD274, TIGIT, and all TGF beta encoding genes in cluster-B verified our hypothesis (Fig. 2f).

### DEGs and functional annotation of the subtypes

**Fig. 3:**
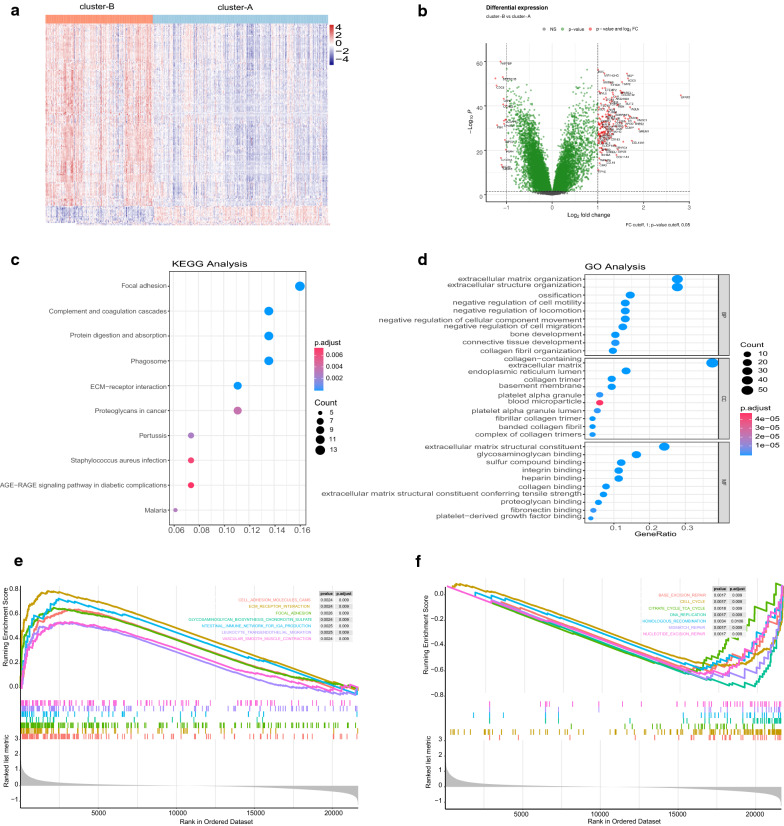
Differentially expressed genes and enrichment analysis. **a**, **b** Heatmap and volcano plot showing the DEG expression between cluster-A and cluster-B. **c**, **d** Top significant terms of KEGG analysis and GO analysis of DEGs between cluster-A and cluster-B. The size of the bubbles denotes the number of genes enriched in the corresponding pathways, and the difference in color represents distinct significance. *BP* biological process, *MF* molecular function, *CC* cellular component. The size of bubbles denotes the number of genes enriched in the corresponding pathway, and the difference in color represents distinct significance. **e**, **f** Results of significantly upregulated pathways and downregulated pathways of “c2.cp.kegg. v7.1. symbols” in MSigDB for the subtypes by GSEA

Next, to discover the underlying gene expression difference between the subtypes, we conducted DEG analysis and identified 178 DEGs, of which 161 genes were upregulated and 17 genes were downregulated in cluster-B. The expression levels of the DEGs are displayed in Fig. 3a, b. We further conducted a functional analysis of the DEGs. KEGG analysis revealed that 161 upregulated genes in cluster-B were significantly involved in the focal adhesion pathway, ECM–receptor interaction pathway, and proteoglycans in cancer pathway (Fig. 3c). GO analysis also indicated their roles in ECM-related processes, such as extracellular structure and matrix organization, glycosaminoglycan binding, and fibronectin-binding (Fig. 3d). We also conducted GSEA for all transcripts ranked by the log2 (fold change) between cluster-A and cluster-B. Similarly, cluster-B was found to have high expression of a gene set related to ECM-related processes and leukocyte transendothelial migration, while cluster-A was related to cell damage repair programs, such as the base excision repair pathway, mismatch repair pathway, and nucleotide excision repair pathway (Fig. 3e, f).

### Construction of the MRS in the GSE39582 dataset and validation in the TCGA and GSE17537 datasets

**Fig. 4:**
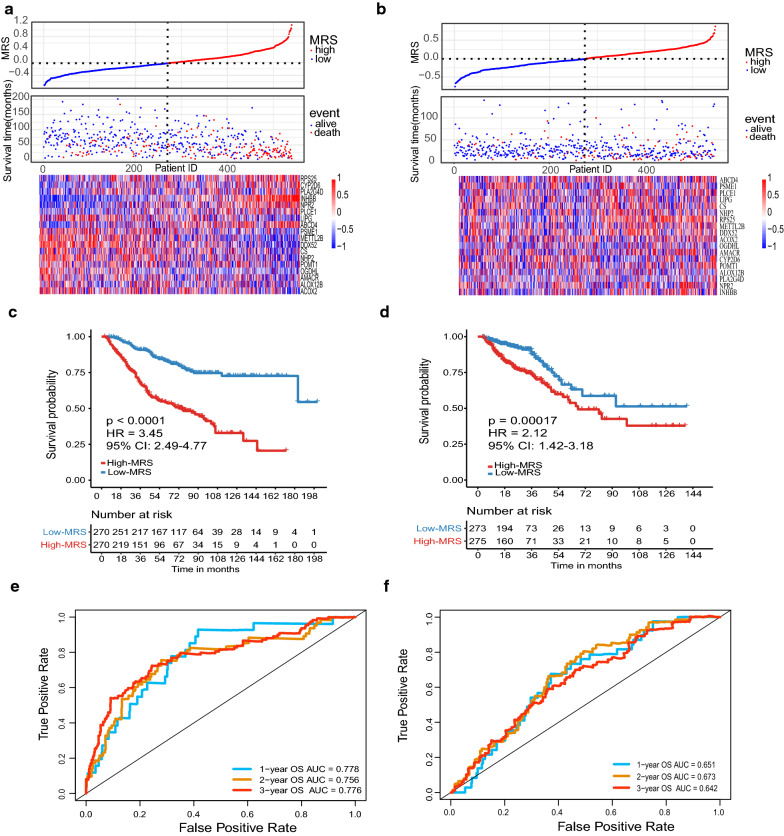
Construction of the metabolic risk score (MRS) in the GSE39582 dataset and validation in the TCGA dataset. **a**, **b** Distributions of the MRS, survival status, and gene expression of 18 genes in the GSE39582 dataset and TCGA dataset. **c**, **d** Kaplan–Meier curves of overall survival of patients in the high- and low-MRS groups in the GSE39582 dataset (**c**) and TCGA dataset (**d**). **e**, **f** ROC curves for predicting 1-, 2-, 3-year overall survival in the GSE39582 dataset (**e**) and TCGA dataset (**f**). *AUC* area under the curve

To develop a robust risk signature for clinical use, we first identified 219 survival-related metabolic genes using univariate Cox regression analysis with a rigorous threshold of *P* < 0.01. Next, we used LASSO penalty regression to build the MRS in GSE39582 via the “glmnet” package (Additional file 10: Figure S3a). We finally identified 18 genes with nonzero coefficients (Additional file 7: Table S7). Both gene expression heatmaps and survival analysis revealed that high expression of LIPG, PSME1, METTL2B, DDX52, CS, NHP2, POMT1, OGDHL, AMACR, ALOX12B, and ACOX2 was correlated with favorable prognosis, whereas high-expressed RPS25, CYP2D6, PLA2G4D, INHBB, NPR2, PLCE1, LIPG, and ABCD4 was correlated with poor prognosis (Fig. 4a, b and Additional file 9: Figure S2; Additional file 11: Figure S4a). Then, the patients were scored using the formula (detailed in Additional file 7: Table S7) and dichotomized into high-MRS and low-MRS groups by the median value of the MRS. In the GSE39582 dataset, patients with high MRS had shorter OS (HR 3.45, 95% CI 2.49–4.77; *P* < 0.0001; Fig. 4c) than those with low MRS, showing that the MRS had a better risk stratification capability than the constructed subtype (Fig. 1c). Similar results were observed in the TCGA dataset (HR 2.12, 95% CI 1.42–3.18; *P* = 0.00017; Fig. 4d) and GSE17537 dataset (HR 2.67, 95% CI 1.01–7.03; *P* = 0.039; Additional file 11: Figure S4b). The survival disadvantage of the high-MRS group was maintained regardless of TNM stage, treatment with chemotherapy, MMR status or microsatellite instability (MSI) status (Additional file 10: Figure S3b–k). ROC analysis was used to evaluate the predictive performance of the MRS in the training cohort and two external cohorts (Fig. 4e, f and Additional file 11: Figure S4c).

### Association between the MRS and clinical immune characteristics

**Fig. 5:**
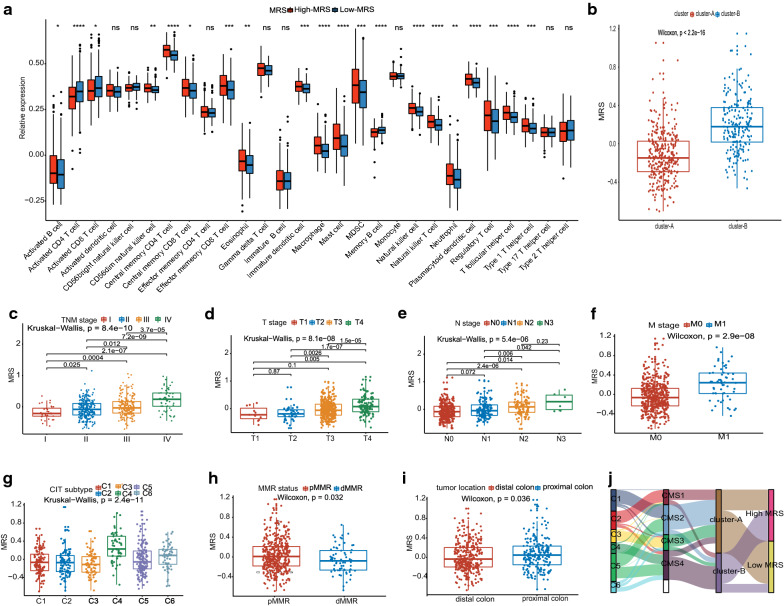
Associations of the MRS with clinical immune characteristics. **a** Immune cell infiltration discrepancy between the high- and low-MRS groups. **b**–**h** Relationships between metabolic subtype (**b**), TNM stage (**c**–**f**), CIT subtype (**g**), MMR status (**h**), and tumor location (**i**) and the MRS. **j** Sankey plot showing the distribution of metabolic subtypes and MRS groups in C1–C6 subtypes and CMS molecular subtypes. *CIT* Cartes d’identité des tumeurs, *MMR* mismatch repair, *dMMR* deficient mismatch repair, *pMMR* proficient mismatch repair, *CMS* consensus molecular subtypes

We further investigated whether there were differences in the abundance of tumor-infiltrating cells between the high- and low-MRS groups. The low-MRS group was characterized by a relatively high infiltration of effector cells, such as activated CD4 T cells and activated CD8 T cells, while the high-MRS group was characterized by a relatively high infiltration of immune-suppressed cells, such as immature dendritic cells, mast cells, macrophages, MDSCs, regulatory T cells and T follicular helper cells (Fig. 5A). Moreover, we confirmed that MRS as a continuous variable was negatively correlated with activated CD4 T cells and activated CD8 T cells but positively correlated with immature dendritic cells, mast cells, MDSCs, T follicular helper cells using Spearman correlation analysis (all *P* < 0.05) (Additional file 12: Figure S5g–l).

We next explored the distribution of the MRS in terms of clinical variables. Higher MRS was found in patients in cluster-B, and in those with advanced TNM stages, the C4C6 subtype, proximal colon cancer, and pMMR status (all *P* values < 0.05, Fig. 5b–i), which have been previously confirmed to have a poor prognosis [18, 31, 32]. No significant difference in MRS were found for BRAF status, KRAS status, TP53 status, sex, CIN status, or CIMP status (Additional file 12: Figure S5a–f). The characteristic changes of individual patients are presented in Fig. 5j.

### Combining the MRS and clinical variables to build a nomogram

**Fig. 6:**
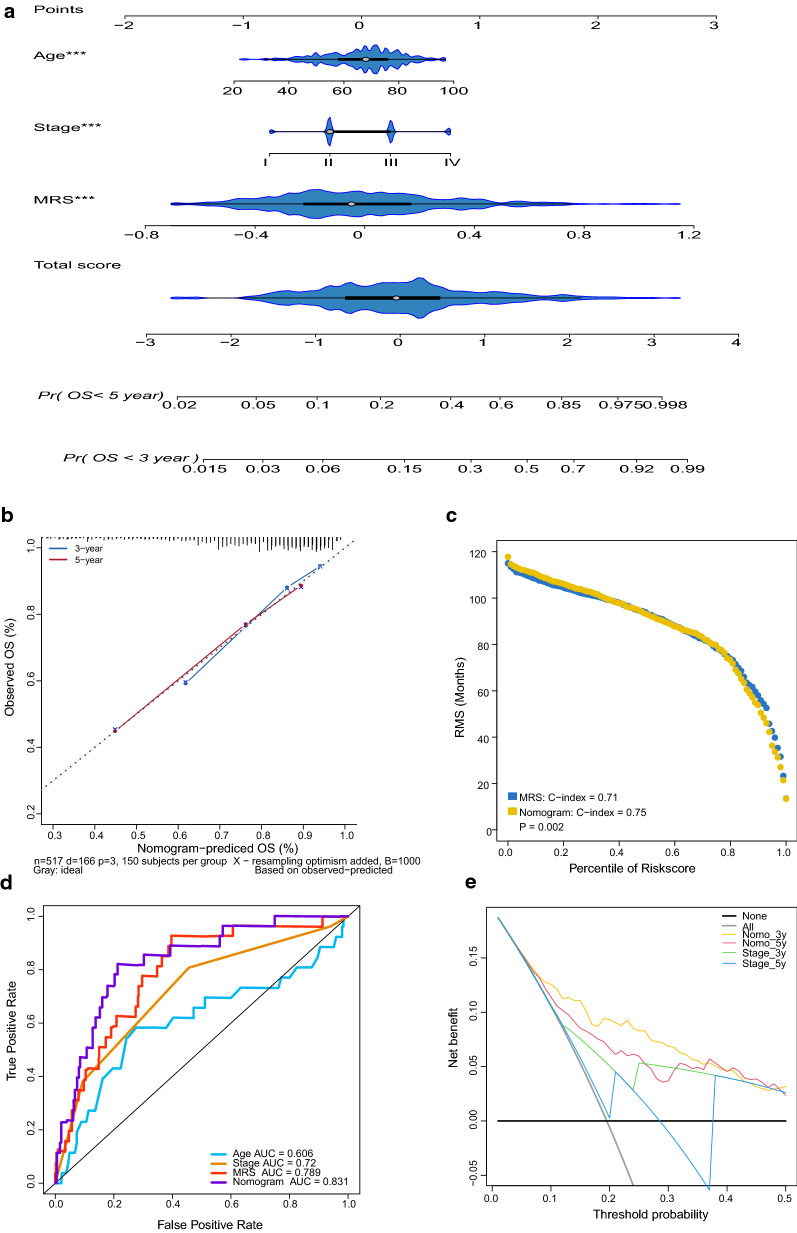
Development of the nomogram in the GSE39582 dataset. **a** Nomogram for predicting the 3-year and 5-year OS of colorectal cancer patients. **b** Calibration plot of the nomogram for predicting 3-year and 5-year OS. **c** RMS curves showing the improvement of the predictive power of the nomogram compared with that of MRS alone. *P* values measure the difference between the two models. **d** ROC curves showing the comparisons of age, stage, MRS, and the nomogram in predicting OS. **e** Decision curves of stage and nomogram in comparing the clinical net benefit

Univariate and multivariate Cox analyses indicated that the MRS could independently predict the OS of CRC patients (Table 1). Then, we combined the MRS with prognostic clinical characteristics to build a nomogram to predict 3-year and 5-year OS (Fig. 6a). The calibration plot indicated that the constructed nomogram performed well when compared with the ideal model in the training, validation and entire cohorts (Fig. 6b, and Additional file 13: Figure S6a; Additional file 14: Figure S7a; Additional file 15: Figure S8a). We also observed a remarkable enhancement in predictive value for the nomogram compared with the MRS alone (nomogram, C-index: 0.75; MRS, C-index: 0.71; *P* = 0.002; Fig. 6c). Similar results were also obtained in the validation cohort and the entire cohort (Additional file 13: Figure S6b; Additional file 14: Figure S7b; Additional file 15: Figure S8b). ROC analysis and DCA also confirmed that the predictive capacity of the nomogram outperformed that of age, stage, and the MRS alone (Fig. 6e, f and Additional file 13: Figure S6c, d, Additional file 14: Figure S7c, d, Additional file 15: Figure S8c, d).

**Table 1:** Univariate and multivariate analyses of the GSE39582 dataset

Variables	Univariate analysis		Multivariate analysis	
	HR (95% CI)	*P*	HR (95% CI)	*P*
Age (years)				
< 68	Ref.		Ref.	
≥ 68	1.44 (1.06–1.94)	0.02	1.51 (1.09–2.10)	0.01
Adjuvant chemotherapy			NE	
No	Ref.			
Yes	1.01 (0.75–1.37)	0.95		
Sex			NE	
Male	Ref.			
Female	0.77 (0.57–1.04)	0.09		
BRAF status			NE	
Wildtype	Ref.			
Mutant	1.05 (0.60–1.81)	0.90		
KRAS status				
Wildtype	Ref.		Ref.	
Mutant	1.39 (1.03–1.88)	0.03	1.38 (1.00–1.89)	0.05
TP53 status			NE	
Wildtype	Ref.			
Mutant	1.18 (0.82–1.68)	0.37		
CIMP status			NE	
Negative	Ref.			
Positive	0.97 (0.63–1.49)	0.90		
CIN status			NE	
Negative	Ref.			
Positive	0.80 (0.54–1.19)	0.26		
MMR status				
pMMR	Ref.		NE	
dMMR	0.79 (0.48–1.30)	0.35		
Tumor location			NE	
Proximal colon	Ref.			
Distal colon	0.91 (0.67–1.23)	0.55		
CIT subtype				
C4C6	Ref.		Ref.	
Others	0.61 (0.44–0.84)	0.002	0.92 (0.64–1.34)	0.68
TNM stage				
I + II	Ref.		Ref.	
III + IV	1.98 (1.45–2.70)	< 0.001	1.52 (1.10–2.11)	0.01
MRS	15.89 (9.97–25.33)	< 0.001	13.59 (8.12–22.72)	< 0.001

*CIMP* CpG island methylator phenotype, *CIN* chromosomal instability, *CIT* d’identité des tumeurs, *MMR* mismatch repair, *dMMR* deficient mismatch repair, *pMMR* proficient mismatch repair, *MRS* metabolic risk score, *NE* not entered

## Discussion

CRC is a heterogeneous disease, with a 5-year survival rate ranging from 90% for patients with localized disease to 14% for those with distant disease [1]. Although widely implemented in clinical practice, the AJCC stage system has been revealed by Martin et al. and his colleagues to have insufficient information for predicting prognosis individually [4].

Metabolic reprogramming, one of the hallmarks of cancer, is closely associated with the growth, invasion, and metastasis of CRC through interactions with stromal cells, immune cells, gut microbiota, and driver mutations [33]. More importantly, studies suggest that metabolic reprogramming may blunt immune responses through T-cell starvation and suppressive metabolite secretion [10, 34, 35]. A pan-cancer study found that the expression patterns of metabolic pathway-related genes could reflect the actual metabolic activities in patients [14]. Therefore, the exploration of metabolism-related subtypes and relevant immune landscapes may reveal metabolic heterogeneity, which may help to explain the survival heterogeneity among CRC patients. Additionally, the development of a prognostic model based on metabolism-related genes could offer novel targets for therapeutic regimens.

Herein, we used a machine learning method to identify two metabolism-related subtypes (cluster-A and cluster-B) with distinct metabolic patterns. Cluster-B exhibited overexpression in immune cells and immune relevant pathways, but worse prognosis than cluster-A. Further analysis revealed that cluster-B also showed overexpression of checkpoint genes and TGF beta related transcripts, which have been confirmed to have immune suppression features [29, 30, 36, 37]. Additionally, we observed a higher stromal score in the cluster-B group, and emerging evidence suggests that a higher stromal population is associated with tumor progression by reshaping antitumor immunity and the responsiveness to immunotherapy [37, 38]. Cluster-B had a closer association with CMS4 subtype, which is characterized by stromal invasion and mesenchymal activation. Overall, cluster-B exhibited a CMS4-like phenotype, and the combination of antimetabolites that target metabolic pathways, such as glycosaminoglycan metabolism and immunotherapy might potentially reverse the immune dysfunction state of patients in this group [34].

Based on the prognostic role of the abovementioned metabolism-related genes we discovered, we attempted to develop an MRS to estimate the individual mortality risk of patients. An 18-gene risk signature was constructed for prognostic prediction for each patient and demonstrated reliable predictive performance in the training, validation and entire cohorts. Since the tumor microenvironment plays a crucial role in the antitumor response [37, 39, 40], we also investigated the abundances of immune cells and stromal cells in the high and low MRS groups. As expected, patients in the low MRS group had a high infiltration of cytotoxic immune cells and relatively low infiltration of immune-suppressed cells, which indicated that this group is more likely to benefit from immunotherapy than its counterpart. Of note, higher MRS was correlated with advanced pathological stage, the C4C6 subtype, pMMR, and proximal colon cancer, and these subgroups have been previously found to have undesirable outcomes [18], which further supports the prognostic value of the MRS. More importantly, multivariate Cox and subgroup analyses indicated that the MRS can be used as a supplement to clinical risk factors to improve risk prediction. Multiple validation methods (i.e., calibration plots, ROC analysis, C-index and decision curve analysis) confirmed that the nomogram incorporating that MRS and clinical risk factors had an improved predictive accuracy, and may serve as a promising tool in individualized risk management.

Most metabolism-related genes included in the MRS have been reported to be associated with cancer. Inhibin subunit beta B (INHBB), one of the miR-34 targets, was found to be related to lymph node metastases and poor survival in primary CRC [41, 42]. NHP2 ribonucleoprotein (NHP2, also termed DKCB2, NHP2P, and NOLA2) is a telomere-related gene, and changes in its coding sequence may be involved in the pathogenesis of familial breast cancer [43]. Oxoglutarate dehydrogenase L (OGDHL) encodes an oxoglutarate dehydrogenase complex subunit and indirectly participates in the process of apoptosis. The inactivation of OGDHL through promoter hypermethylation may result in the downregulated expression of the gene in CRC, thereby impairing its function of inducing apoptosis [44]. PLA2G4D, a member of the phospholipase A2 enzyme family, was defined as a recurrently mutated gene that leads to the pathogenesis of splenic marginal zone lymphoma [45]. Phospholipase C epsilon 1 (PLCE1), has been found to promote angiogenesis and proliferation in esophageal squamous cell carcinoma by activating the NF-κB signaling pathway and inducing VEGF-C/Bcl-2 expression [46]. PSME1, also known as PA28A, has been identified as negatively regulated in the Wnt pathway and serves as a preferable prognostic marker in CRC [47]. Our results also showed similar findings. However, other metabolism-related genes included in the MRS have not been associated with outcome in cancer, and how they regulate prognosis should be explored in vitro and in vivo.

Some limitations must be underscored in the current study. First, although we included a total of 1142 patients from both microarray and RNA-seq platforms, which indicates that the conclusion may be highly reliable, robust, and immune from platform bias, the MRS should be further validated in a prospective study due to the retrospective nature of the current study. Moreover, our analysis is based on bioinformatic analysis of tumor samples collected from public datasets, and additional experimental studies and validation of the signatures on clinical specimens are needed in further research. Last, the mechanisms underlying metabolic regulation and CRC prognosis of several metabolism-related genes included in the MRS needed to be explored.

## Conclusions

In summary, we identified two metabolism-related subtypes of CRC, and further evaluated the difference in immune networks and signaling pathways underlying the subtypes, which provides more insights into the relationship between tumor metabolism and immunity and more evidence for the combination of anti-metabolites and immunotherapy to enhance antitumor immunity. Additionally, the MRS comprising 18 metabolism-related genes was proposed and demonstrated to have remarkable predictive value. The promising prognostic accuracy of the model may facilitate individualized prognosis management and personalized therapeutic intervention. Combining clinical characteristics and the risk signature further improved the predictive performance in CRC.

## Supplementary Information

**Additional file 1: Table S1.** Details of the baseline characteristics of the patients in the GSE39582 dataset.

**Additional file 2: Table S2.** Details of the baseline characteristics of the patients in the TCGA CRC dataset.

**Additional file 3: Table S3.** Details of the baseline characteristics of the patients in the GSE17537 dataset.

**Additional file 4: Table S4.** Twenty-eight immune cells and corresponding gene signatures.

**Additional file 5: Table S5.** Immune-related pathways and corresponding gene signatures.

**Additional file 6: Table S6.** Survival-related metabolic genes used for unsupervised clustering in the GSE39582 dataset.

**Additional file 7: Table S7.** Metabolic pathways and LASSO coefficients of candidate genes used to build the MRS.

**Additional file 8: Figure S1.** The result of individualized consensus molecular subtypes estimated by the “CMScaller” R package.

**Additional file 9: Figure S2.** Kaplan–Meier curves of overall survival for 18 genes when divided into high expression and low expression groups according to the optimal cutoff determined by the “survminer” R package.

**Additional file 10: Figure S3.** LASSO model and subgroup analysis of OS between the high- and low-MRS group in the GSE39582 and TCGA datasets.

**Additional file 11: Figure S4.** Validation of the MRS in the GSE17537 dataset.

**Additional file 12: Figure S5.** Spearman correlations between immune cells and the MRS and distribution of the MRS among various clinical variables.

**Additional file 13: Figure S6.** Validation of the nomogram in the TCGA CRC dataset.

**Additional file 14: Figure S7.** Validation of the nomogram in the GSE17537 dataset.

**Additional file 15: Figure S8.** Validation of the nomogram in the entire dataset.

## Data Availability

The datasets analyzed in this study are available in public databases.

## Abbreviations

CRC: Colorectal cancer
GEO: Gene Expression Omnibus
TCGA: The Cancer Genome Atlas
CMS: Consensus molecular subtype
TPM: Transcripts per kilobase million
MSigDB: Molecular Signatures Database
ssGSEA: Single sample gene set enrichment analysis
DEG: Differentially expressed gene
KEGG: Kyoto Encyclopedia of Genes and Genomes
GO: Gene Ontology
GSEA: Gene set enrichment analysis
LASSO: Least absolute shrinkage and selection operator
MRS: Metabolic risk score
ROC: Receiver operating characteristic
C-index: Concordance index
RMS: Restricted mean survival
DCA: Decision-curve analysis
OS: Overall survival
RFS: Relapse-free survival
CI: Confidence interval
AJCC: American Joint Committee on Cancer
CIMP: CpG island methylator phenotype
CIN: Chromosomal instability
CIT: Cartes d’ identité des tumeurs
MMR: Mismatch repair
dMMR: Deficient mismatch repair
pMMR: Proficient mismatch repair
MSI: Microsatellite instability
MSS: Microsatellite stability
MDSC: Myeloid-derived suppressor cell
