# Supplementary material for: Molecular subtype identification and prognosis stratification by a metabolism-related gene expression signature in colorectal cancer
Source: J Transl Med. 2021 Jun 30;19:279. doi: 10.1186/s12967-021-02952-w (PMC8244251; doi:10.1186/s12967-021-02952-w)
Supplement: Supplementary file 1 — Additional file 1: Table S1. Details of the baseline characteristics of the patients in the GSE39582 dataset. [file 12967_2021_2952_MOESM1_ESM.docx]

Table S1. Details of the baseline characteristics of the patients in the GSE39582 dataset.

| Characteristics | GSE39582 dataset |
| --- | --- |
| No. of patients | 540 |
| Age, median, IQR (years) | 68 (58-76) |
| Follow-up, median(months) | 53 (30-81) |
| Sex (%) |  |
| male | 295 (54.63) |
| female | 245 (45.37) |
| Adjuvant Chemotherapy (%) |  |
| Yes | 233 (43.15) |
| No | 294 (54.44) |
| Unknown | 13 (2.41) |
| Chemotherapy agent (%) |  |
| 5FU | 79 (14.63) |
| FOLFIRI | 11 (2.04) |
| FOLFOX | 23 (4.26) |
| FUFOL | 51(9.44) |
| Other | 3 (0.55) |
| Unknown | 373 (69.08) |
| Tumor location (%) |  |
| Proximal colon | 212 (39.26) |
| Distal colon | 328 (60.74) |
| CIMP status (%) |  |
| Positive | 83 (15.37) |
| Negative | 388 (71.85) |
| Unknown | 69 (12.78) |
| CIN status (%) |  |
| Positive | 340 (62.96) |
| Negative | 100 (18.52) |
| Unknown | 100 (18.52) |
| TP53 status (%) |  |
| Mutant | 184 (34.07) |
| Wildtype | 156 (28.89) |
| Unknown | 200 (37.04) |
| BRAF status (%) |  |
| Mutant | 45 (8.33) |
| Wildtype | 441 (81.67) |
| Unknown | 54 (10.00) |
| KRAS status (%) |  |
| Mutant | 206 (38.15) |
| Wildtype | 314 (58.15) |
| Unknown | 20 (3.70) |
| CIT subtype (%) |  |
| C1 | 113 (20.93) |
| C2 | 97 (17.96) |
| C3 | 68 (12.59) |
| C4 | 57 (10.55) |
| C5 | 147 (27.22) |
| C6 | 58 (10.75) |
| MMR status (%) |  |
| dMMR | 68 (12.59) |
| pMMR | 426 (78.89) |
| Unknown | 46 (8.52) |
| T stage (%) |  |
| T1 | 11 (2.04) |
| T2 | 42 (7.78) |
| T3 | 352 (65.18) |
| T4 | 115 (21.30) |
| Unknown | 20 (3.70) |
| N stage (%) |  |
| N0 | 286 (52.97) |
| N+ | 6 (1.11) |
| N1 | 127 (23.52) |
| N2 | 95 (17.59) |
| N3 | 6 (1.11) |
| Unknown | 20 (3.70) |
| M stage (%) |  |
| M0 | 462 (85.56) |
| M1 | 56 (10.37) |
| Mx | 2 (0.37) |
| Unknown | 20 (3.70) |
| TNM stage (%) |  |
| Stage I | 32 (5.93) |
| Stage II | 241 (44.63) |
| Stage III | 189 (35.00) |
| Stage IV | 56 (10.37) |
| Unknown | 22 (4.07) |

Abbreviations: IQR, interquartile range; 5FU, 5- fluorouracil; FOLFIRI, folinic acid, FU, and irinotecan; FOLFOX, folinic acid, FU, and oxaliplatin; FUFOL, FU and leucovorin; CIMP, CpG island methylator phenotype; CIN, chromosomal instability; CIT, Cartes d’ identité des tumeurs; MMR, mismatch repair; dMMR, deficient mismatch repair; pMMR, proficient mismatch repair. Unknown, unavailable data.
