# Supplementary material for: Molecular subtype identification and prognosis stratification by a metabolism-related gene expression signature in colorectal cancer
Source: J Transl Med. 2021 Jun 30;19:279. doi: 10.1186/s12967-021-02952-w (PMC8244251; doi:10.1186/s12967-021-02952-w)
Supplement: Supplementary file 2 — Additional file 2: Table S2. Details of the baseline characteristics of the patients in the TCGA CRC dataset. [file 12967_2021_2952_MOESM2_ESM.docx]

Table S2. Details of the baseline characteristics of the patients in the TCGA CRC dataset.

| characteristic | TCGA CRC dataset |
| --- | --- |
| No. of patients | 548 |
| Age, median, IQR (year) | 68(58-75) |
| Follow-up, median(month) | 24.18 (14.41,36.92) |
| Radiotherapy (%) |  |
| Yes | 37(6.75) |
| No | 468 (85.40) |
| Not reported | 43(7.85) |
| Prior malignancy (%) |  |
| Yes | 68 (12.41) |
| No | 480 (87.59) |
| Sex (%) |  |
| male | 302(55.11) |
| female | 246 (44.89) |
| Race (%) |  |
| Asian | 12 (2.19) |
| Black or African American | 63(11.50) |
| White | 275(50.18) |
| Not reported | 198 (36.13) |
| MSI status (%) |  |
| Indeterminate | 3 (0.55) |
| MSS | 360 (65.69) |
| MSI-L | 87 (15.88) |
| MSI-H | 70 (12.77) |
| Unknown | 28 (5.11) |
| T stage (%) |  |
| Tis | 1 (0.18) |
| T1 | 17 (3.10) |
| T2 | 93 (16.97) |
| T3 | 377 (68.80) |
| T4 | 59 (10.77) |
| Unknown | 1(0.18) |
| N stage (%) |  |
| N0 | 309 (56.39) |
| N1 | 135 (24.64) |
| N2 | 101 (18.43) |
| Nx | 2 (0.36) |
| Unknown | 1 (0.18) |
| M stage (%) |  |
| M0 | 410 (74.82) |
| M1 | 72 (13.14) |
| Mx | 59 (10.77) |
| Unknown | 7 (1.27) |
| TNM stage (%) |  |
| Stage I | 83(15.15) |
| Stage II | 187(34.12) |
| Stage III | 134(24.45) |
| Stage IV | 72(13.14) |
| Unknown | 72(13.14) |

Abbreviation: IQR, interquartile range; CRC, colorectal cancer; MSI, microsatellite instability; IQR, interquartile range. Nx, the status of regional lymph nodes cannot be evaluated. Mx, the status of distal metastasis cannot be evaluated.
