# Supplementary material for: Molecular subtype identification and prognosis stratification by a metabolism-related gene expression signature in colorectal cancer
Source: J Transl Med. 2021 Jun 30;19:279. doi: 10.1186/s12967-021-02952-w (PMC8244251; doi:10.1186/s12967-021-02952-w)
Supplement: Supplementary file 3 — Additional file 3: Table S3. Details of the baseline characteristics of the patients in the GSE17537 dataset. [file 12967_2021_2952_MOESM3_ESM.docx]

Table S3. Details of the baseline characteristics of the patients in the GSE17537 dataset.

| Characteristics | GSE17537 dataset |
| --- | --- |
| No. of patients | 54 |
| Age, median, IQR (year) | 62(54.25-72) |
| Follow-up, median(month) | 51.16(32.66-60.03) |
| Sex (%) |  |
| male | 26(48.14) |
| female | 28(51.86) |
| Ethnicity (%) |  |
| black | 4 (7.41) |
| caucasian | 49 (90.74) |
| hispanic | 1 (1.85) |
| Grade (%) |  |
| WD | 1 (1.85) |
| MD | 25 (46.30) |
| MPD | 7 (12.96) |
| PD | 3 (5.56) |
| Unknown | 18 (33.33) |
| TNM stage (%) |  |
| Stage I | 4 (7.41) |
| Stage II | 15 (27.78) |
| Stage III | 19 (35.18) |
| Stage IV | 16 (29.63) |

Abbreviations: IQR, interquartile range; WD, Well-differentiated; MD, Median- differentiated; MPD, Median to poor differentiated; PD, Poor-differentiated.
