# Supplementary material for: Molecular subtype identification and prognosis stratification by a metabolism-related gene expression signature in colorectal cancer
Source: J Transl Med. 2021 Jun 30;19:279. doi: 10.1186/s12967-021-02952-w (PMC8244251; doi:10.1186/s12967-021-02952-w)
Supplement: Supplementary file 7 — Additional file 7: Table S7. Metabolic pathways and LASSO coefficients of candidate genes used to build the MRS. [file 12967_2021_2952_MOESM7_ESM.docx]

**Table S7. Metabolic pathways and LASSO coefficients of candidate genes used to build the MRS.**

| Metabolic genes | Metabolic pathway | LASSO coefficients |
| --- | --- | --- |
| NPR2 | PURINE_METABOLISM | 0.001147931 |
| ALOX12B^¶^ | ARACHIDONIC_ACID_METABOLISM | -0.001714569 |
| ALOX12B^¶^ | METABOLISM_OF_LIPIDS | -0.001714569 |
| ALOX12B^¶^ | FATTY_ACID_METABOLISM | -0.001714569 |
| ABCD4 | DEFECTS_IN_VITAMIN_AND_COFACTOR_METABOLISM | 0.103628320 |
| OGDHL | TRYPTOPHAN_METABOLISM | -0.093368820 |
| LIPG | GLYCEROLIPID_METABOLISM | -0.021189816 |
| PLCE1 | INOSITOL_PHOSPHATE_METABOLISM | 0.062357273 |
| ALOX12B¶ | HISTIDINE_METABOLISM | -0.001714569 |
| CS^¶^ | GLYOXYLATE_AND_DICARBOXYLATE_METABOLISM | -0.002864414 |
| CS^¶^ | PYRUVATE_METABOLISM_AND_CITRIC_ACID_TCA_CYCLE | -0.002864414 |
| CYP2D6^¶^ | DRUG_METABOLISM_CYTOCHROME_P450 | 0.004665854 |
| CYP2D6^¶^ | METABOLISM_OF_LIPIDS | 0.004665854 |
| ACOX2^¶^ | BILE_ACID_AND_BILE_SALT_METABOLISM | -0.056701244 |
| ACOX2^¶^ | PEROXISOMAL_LIPID_METABOLISM | -0.056701244 |
| ACOX2^¶^ | METABOLISM_OF_LIPIDS | -0.056701244 |
| ACOX2^¶^ | METABOLISM_OF_STEROIDS | -0.056701244 |
| ACOX2^¶^ | FATTY_ACID_METABOLISM | -0.056701244 |
| AMACR^¶^ | BILE_ACID_AND_BILE_SALT_METABOLISM | -0.004773017 |
| AMACR^¶^ | PEROXISOMAL_LIPID_METABOLISM | -0.004773017 |
| AMACR^¶^ | METABOLISM_OF_LIPIDS | -0.004773017 |
| AMACR¶ | METABOLISM_OF_STEROIDS | -0.004773017 |
| PLA2G4D | METABOLISM_OF_LIPIDS | 0.012935257 |
| PSME1 | METABOLISM_OF_POLYAMINES | -0.085578720 |
| RPS25 | DISEASES_OF_METABOLISM | 0.031996003 |
| NHP2 | METABOLISM_OF_RNA | -0.016833515 |
| DDX52 | METABOLISM_OF_RNA | -0.037035969 |
| INHBB | PEPTIDE_HORMONE_METABOLISM | 0.198098959 |
| POMT1 | DISEASES_OF_METABOLISM | -0.015210631 |
| METTL2B^¶^ | HISTIDINE_METABOLISM | -0.051330997 |
| METTL2B^¶^ | TYROSINE_METABOLISM | -0.051330997 |
| METTL2B^¶^ | SELENOAMINO_ACID_METABOLISM | -0.051330997 |

Note: ¶ represents the gene that map to several metabolic pathways.

The detail of formular of MRS is:

MRS = (0.001147931) * NPR2 + (-0.051330997) * METTL2B +(-0.093368820) * OGDHL +(-0.021189816) * LIPG + (0.062357273) * PLCE1 +(-0.001714569) * ALOX12B +(-0.002864414) * CS + (0.004665854) * CYP2D6 + (-0.056701244) * ACOX2 +( -0.004773017) * AMACR +(0.103628320) * ABCD4 + (0.012935257) * PLA2G4D + (-0.085578720) * PSME1 + (0.031996003) * RPS25 + (-0.016833515) * NHP2 + (-0.037035969) * DDX52 + (0.198098959) *INHBB + (-0.015210631) * POMT1
