# Supplementary figures and images for: Molecular subtype identification and prognosis stratification by a metabolism-related gene expression signature in colorectal cancer
Source: J Transl Med. 2021 Jun 30;19:279. doi: 10.1186/s12967-021-02952-w (PMC8244251; doi:10.1186/s12967-021-02952-w)

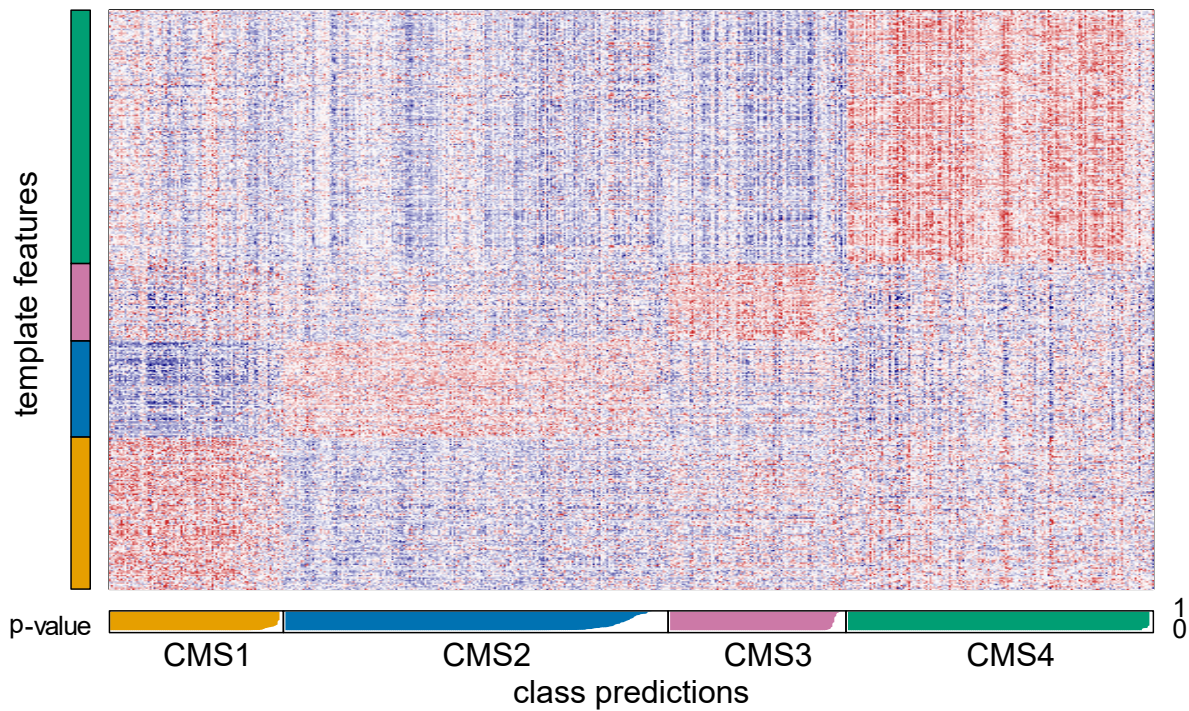

Supplement: Supplementary file 8 — Additional file 8: Figure S1. The result of individualized consensus molecular subtypes estimated by the “CMScaller” R package. [file 12967_2021_2952_MOESM8_ESM.pdf]

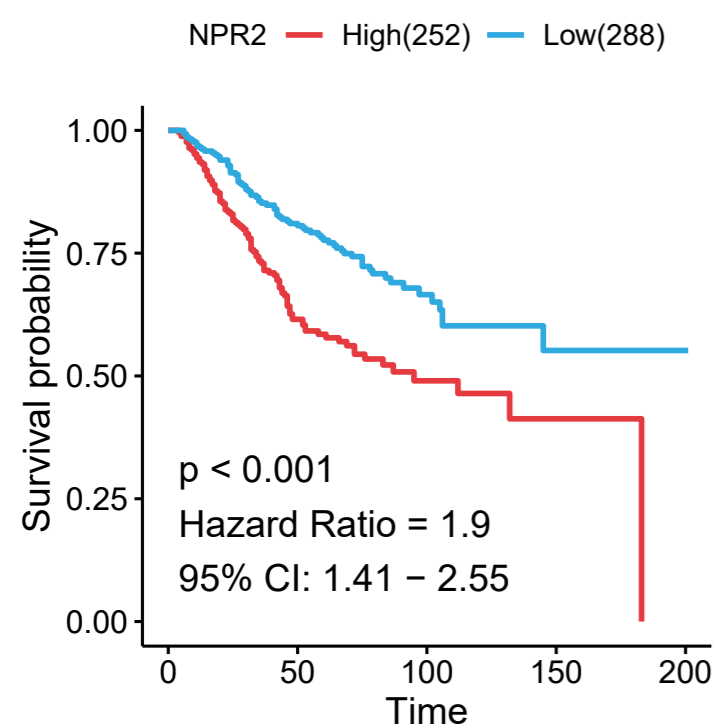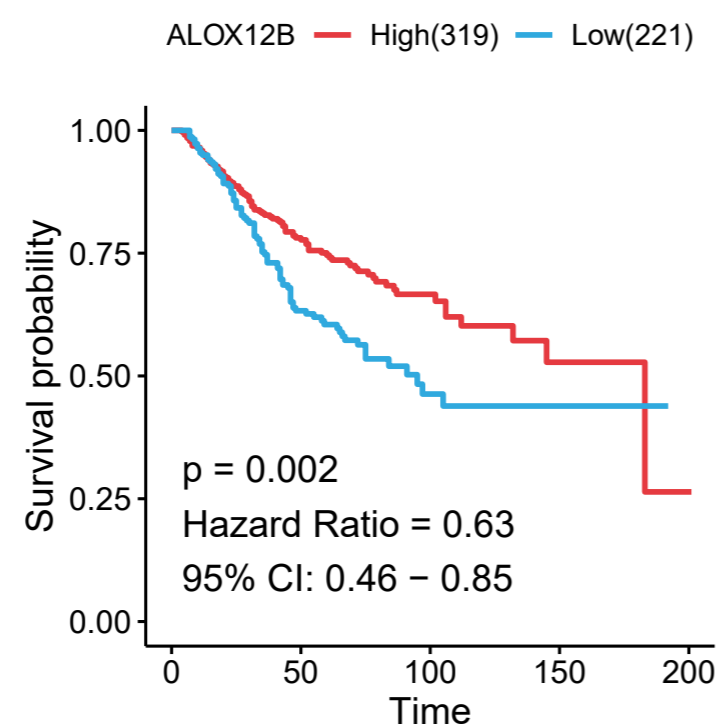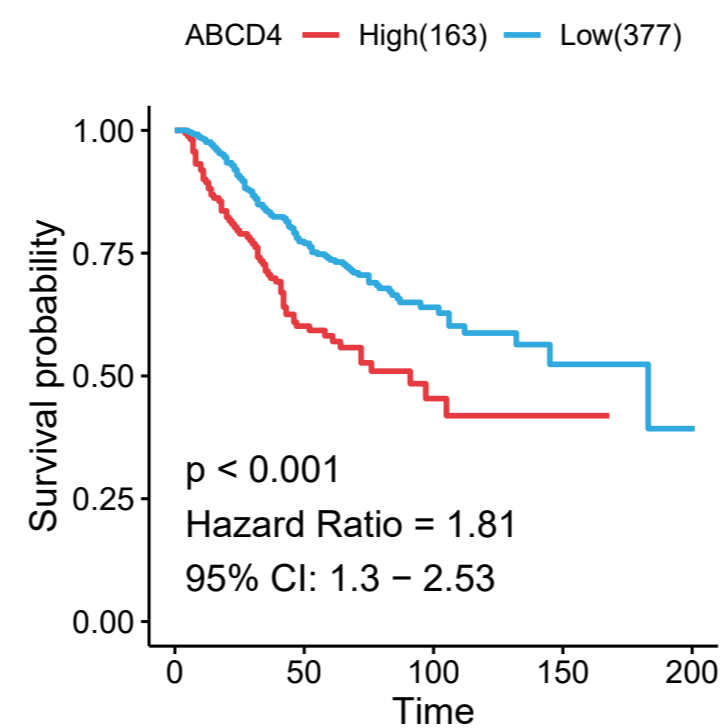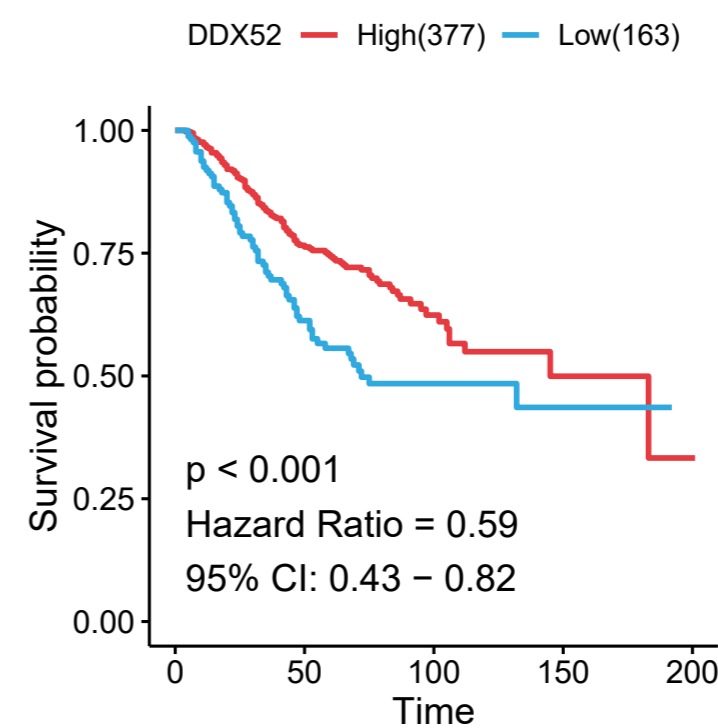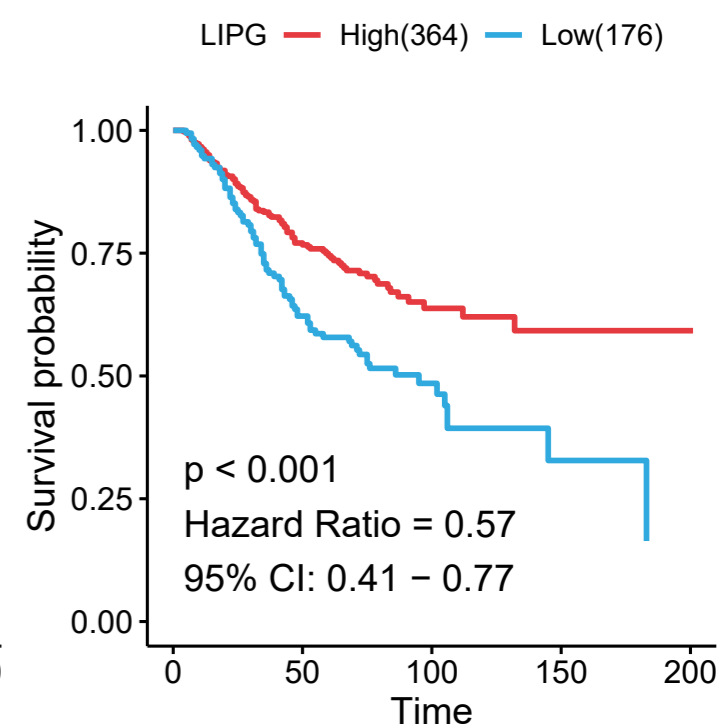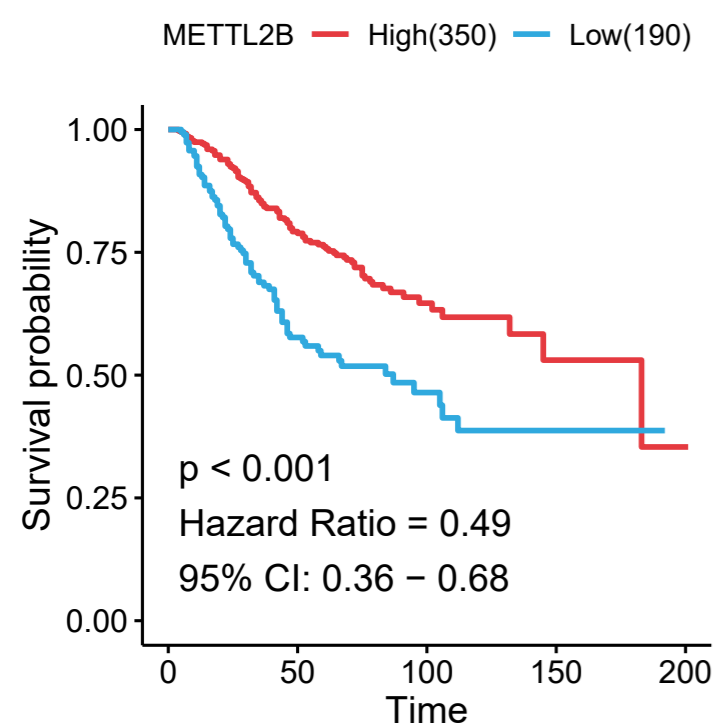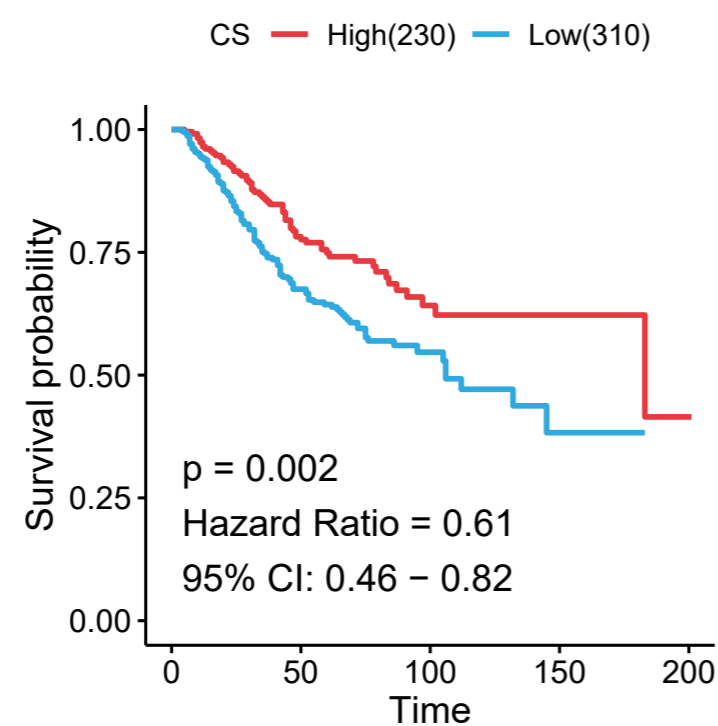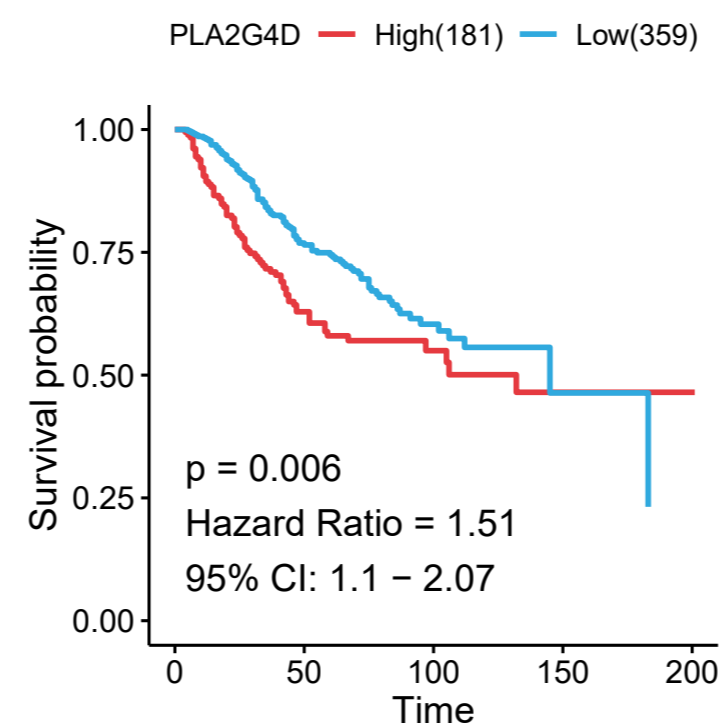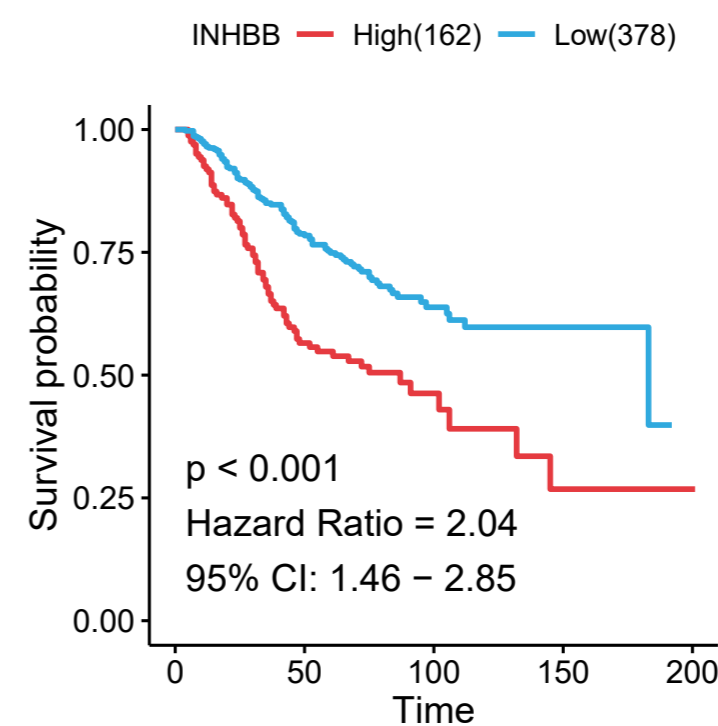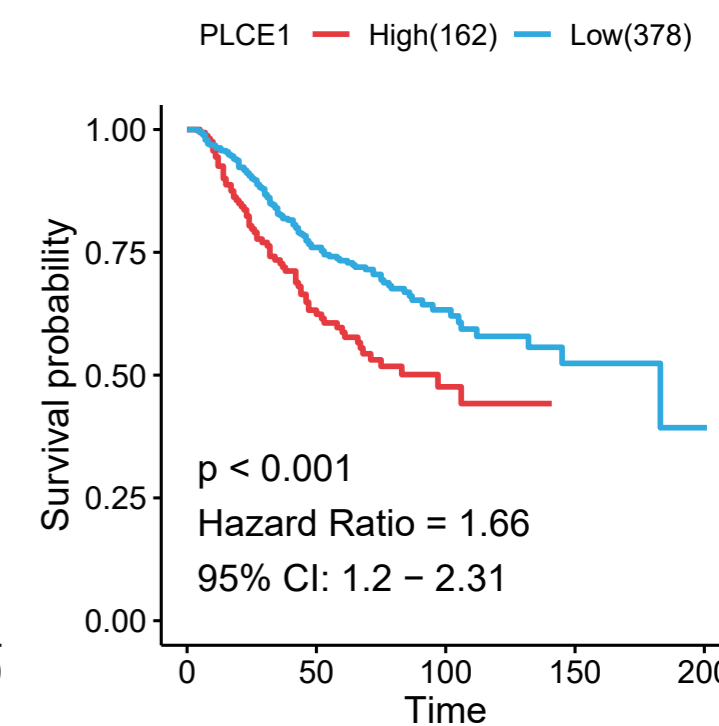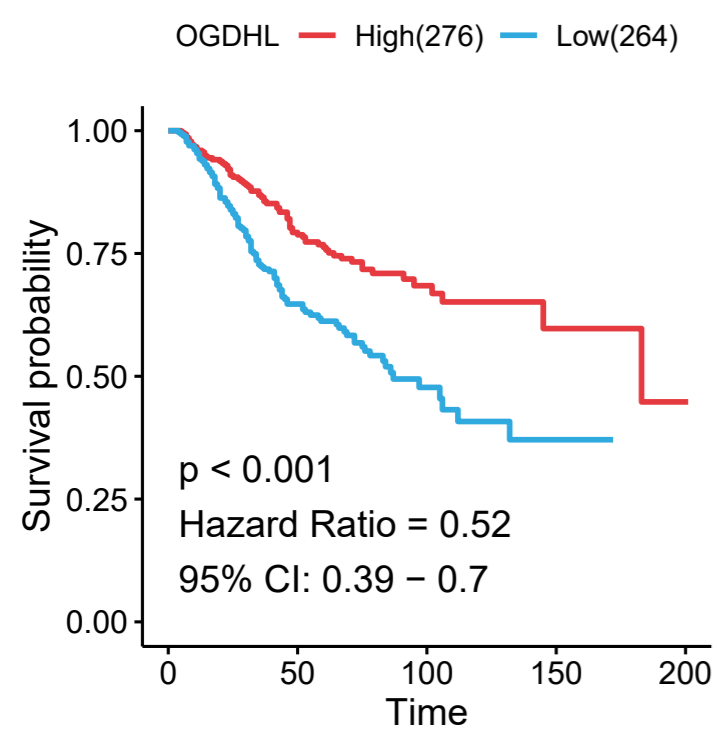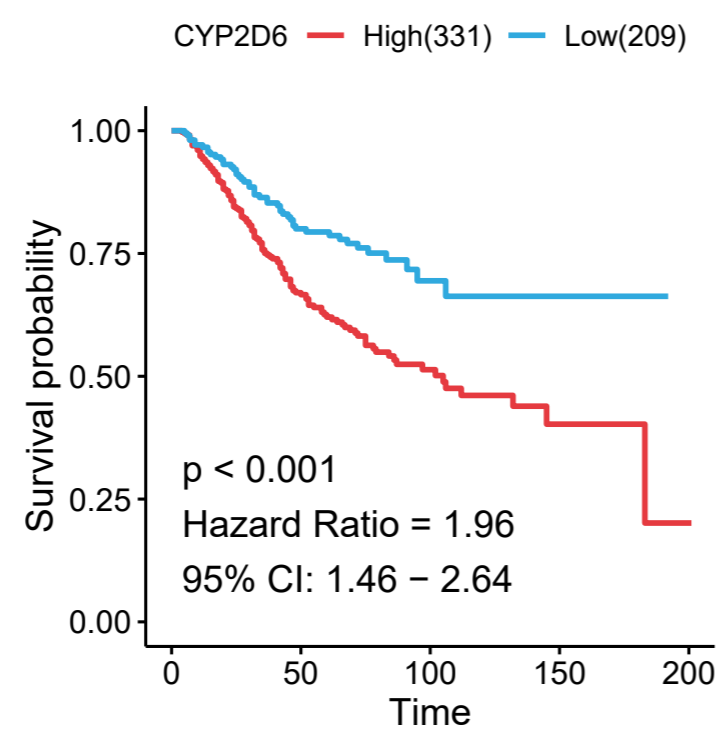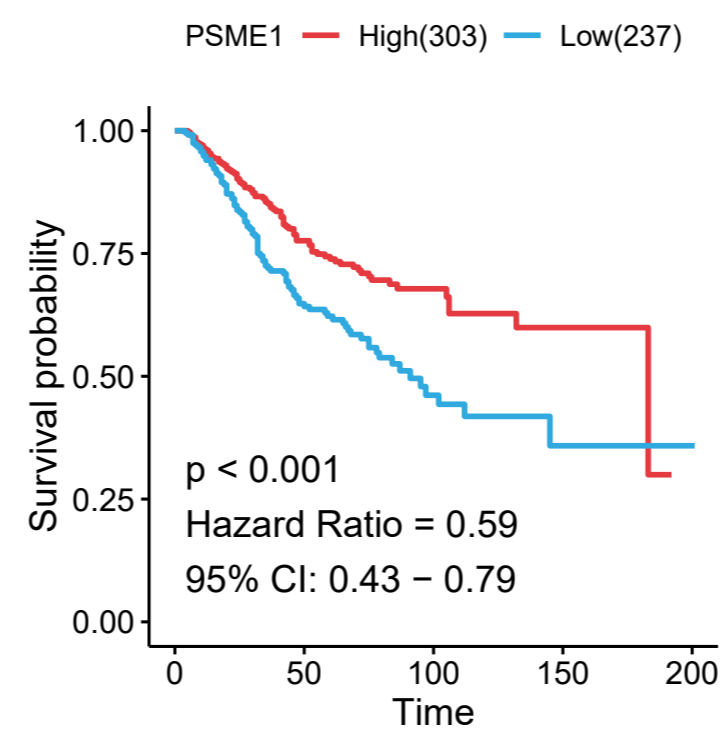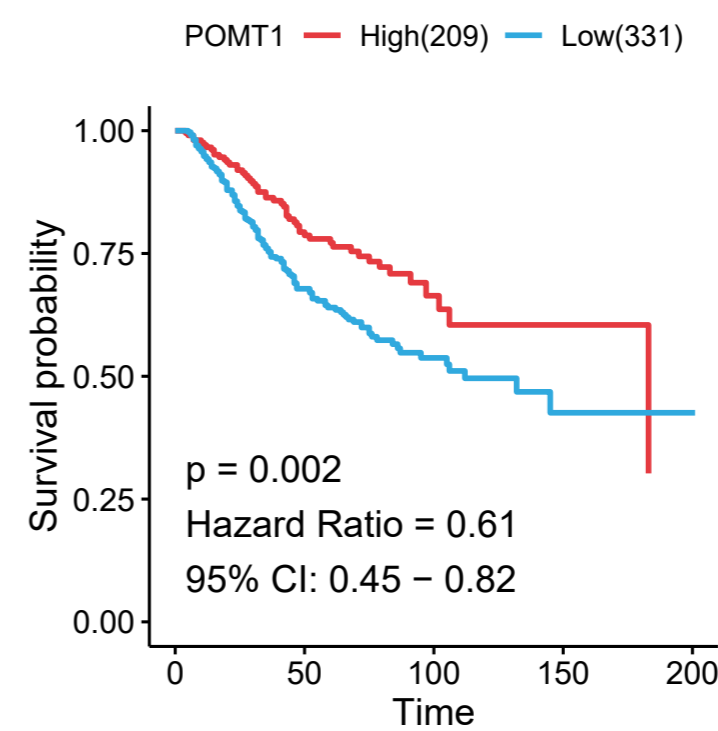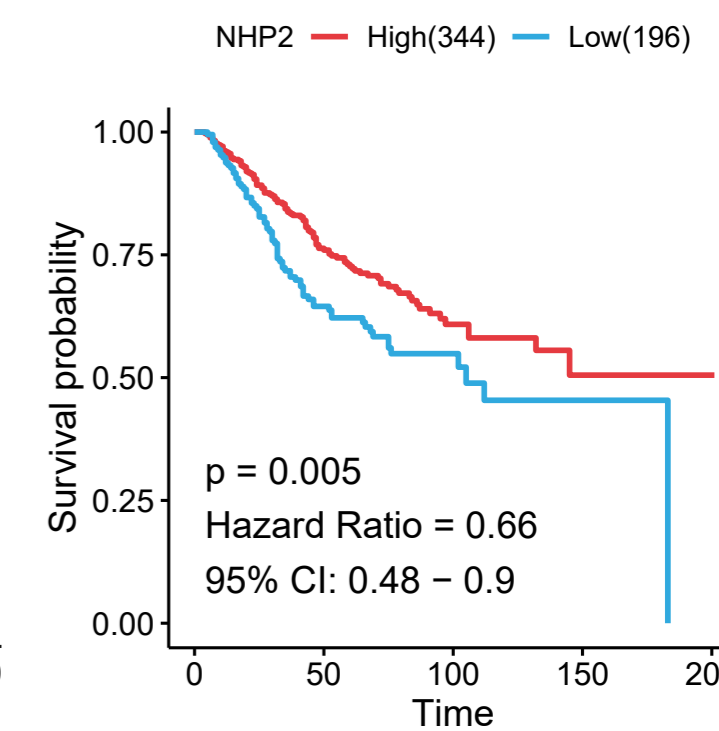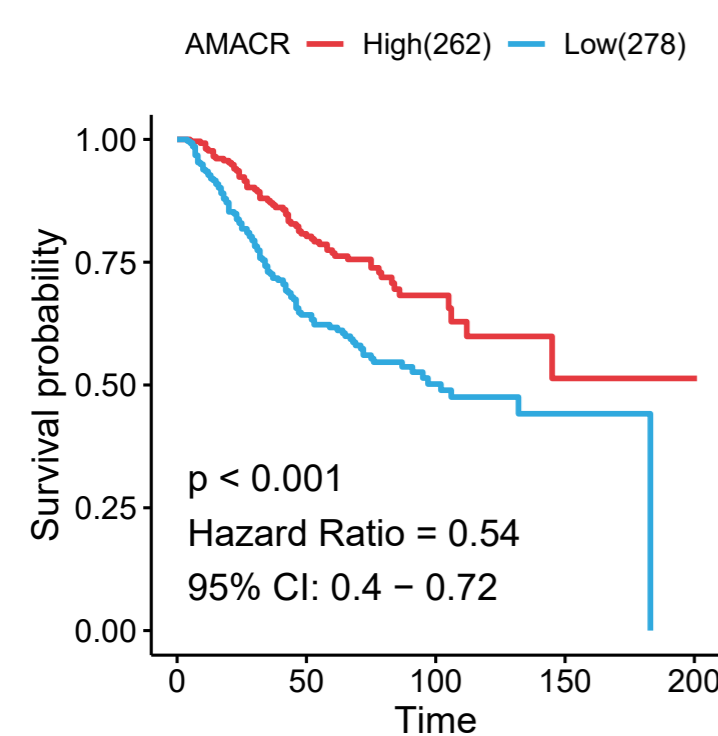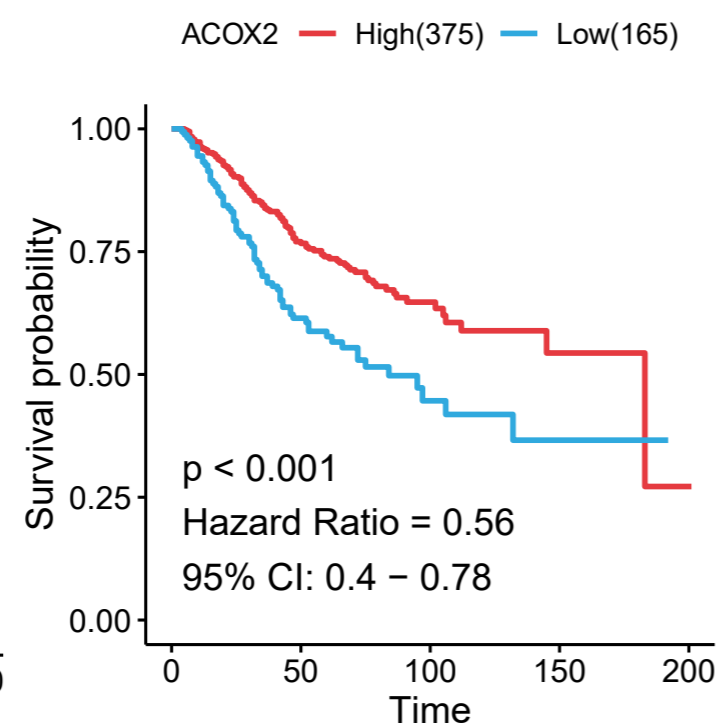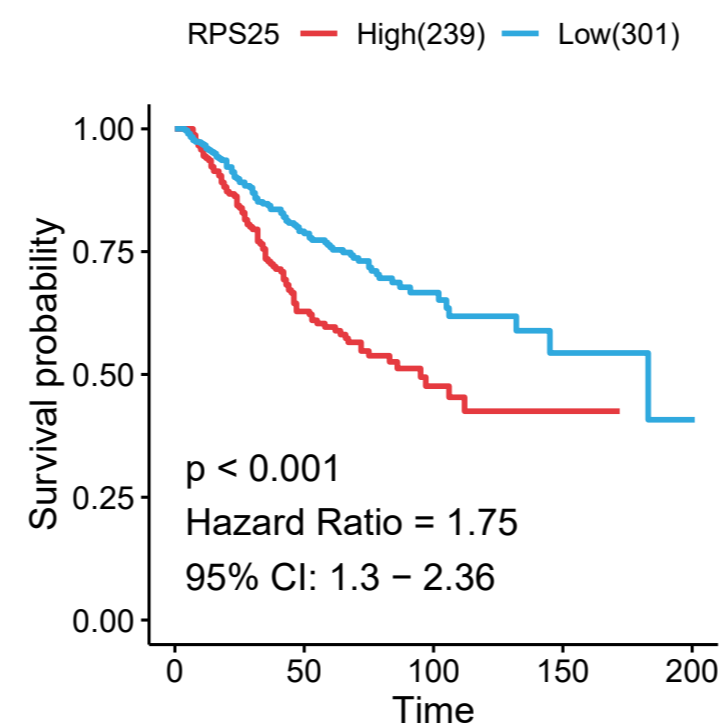

Supplement: Supplementary file 9 — Additional file 9: Figure S2. Kaplan–Meier curves of overall survival for 18 genes when divided into high expression and low expression groups according to the optimal cutoff determined by the “survminer” R package. [file 12967_2021_2952_MOESM9_ESM.pdf]

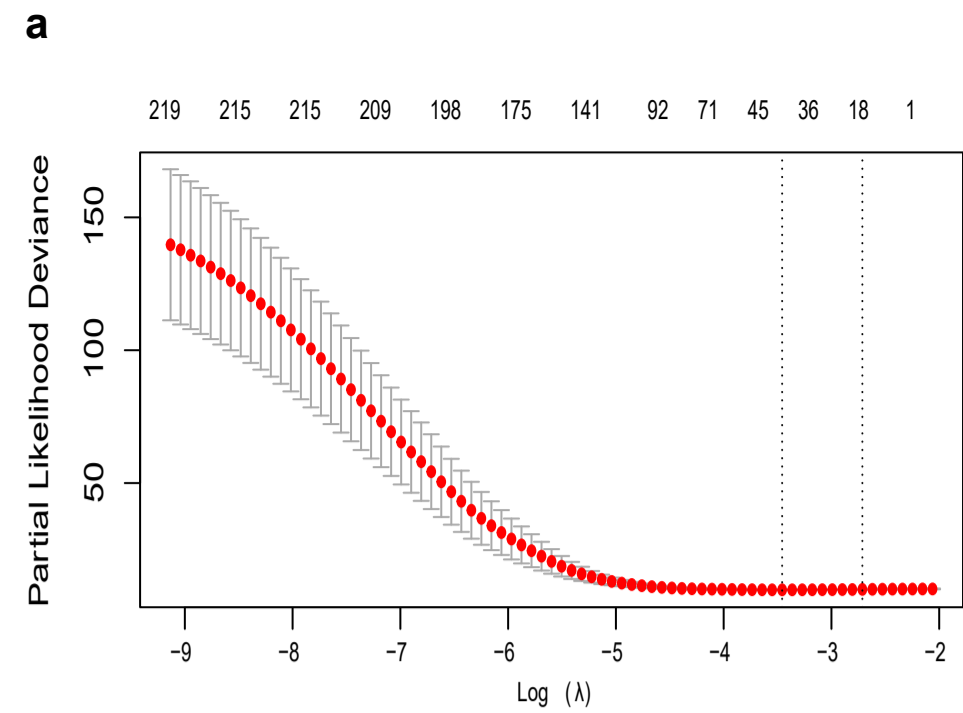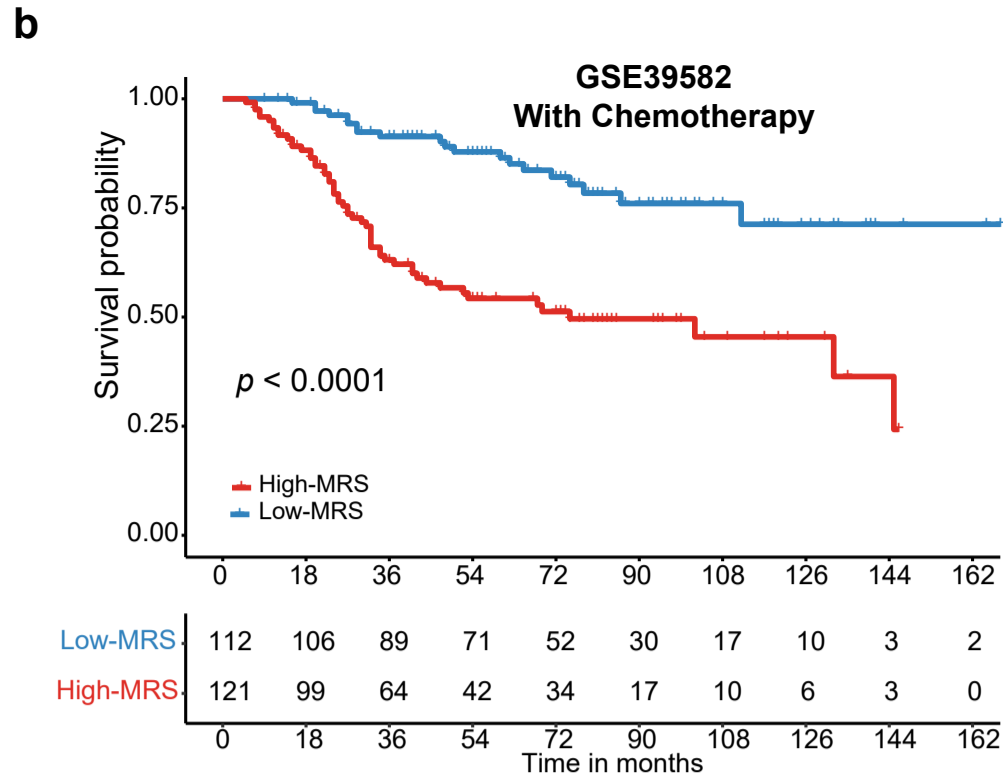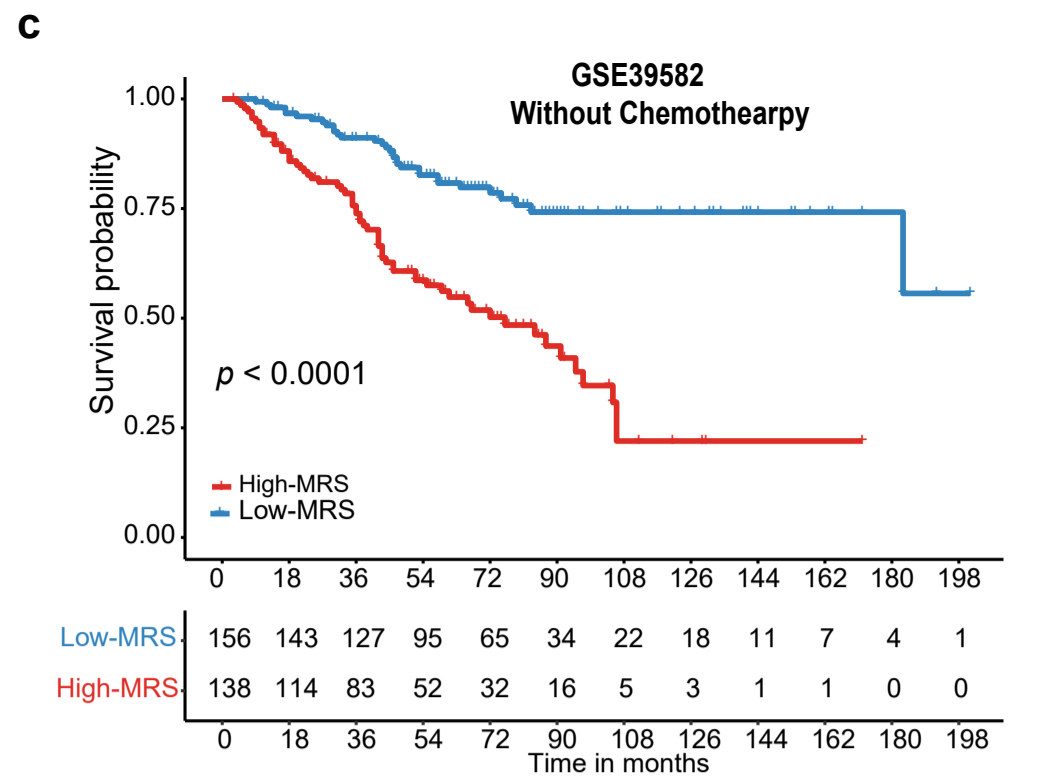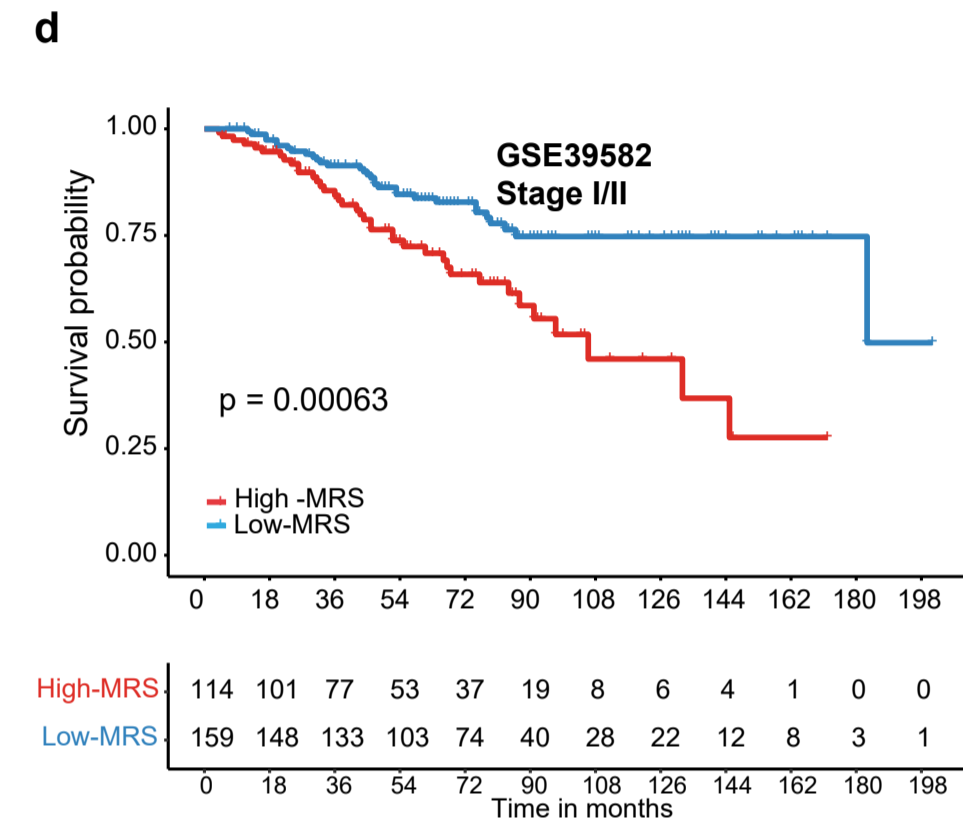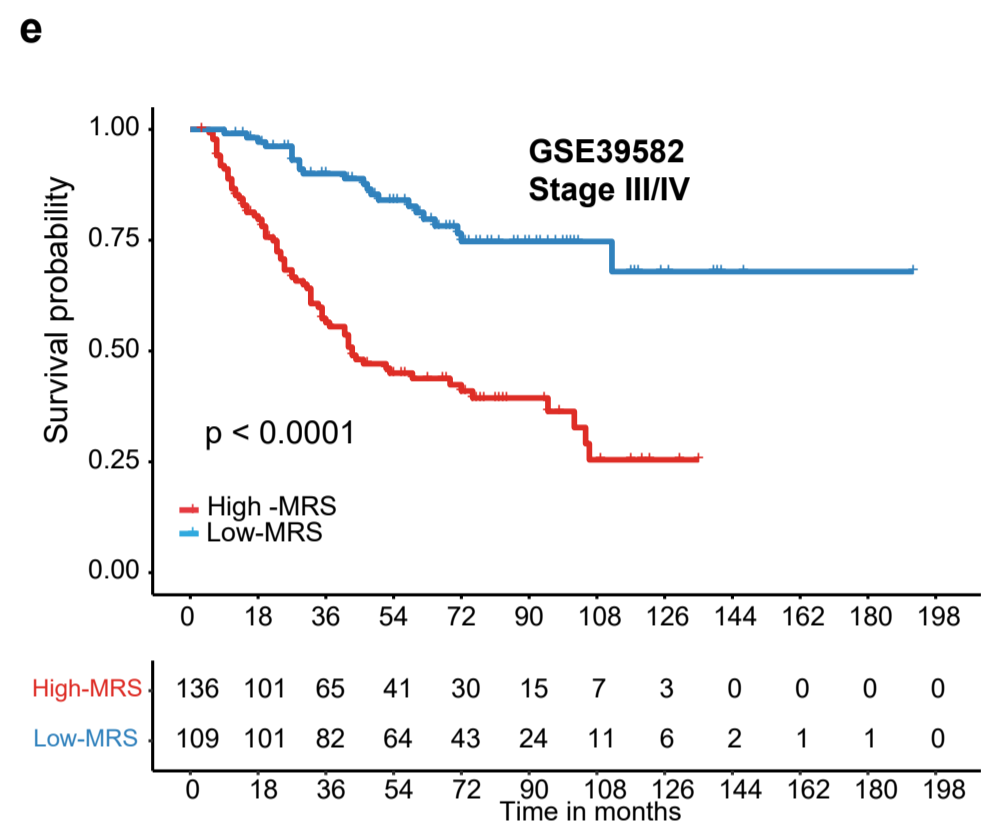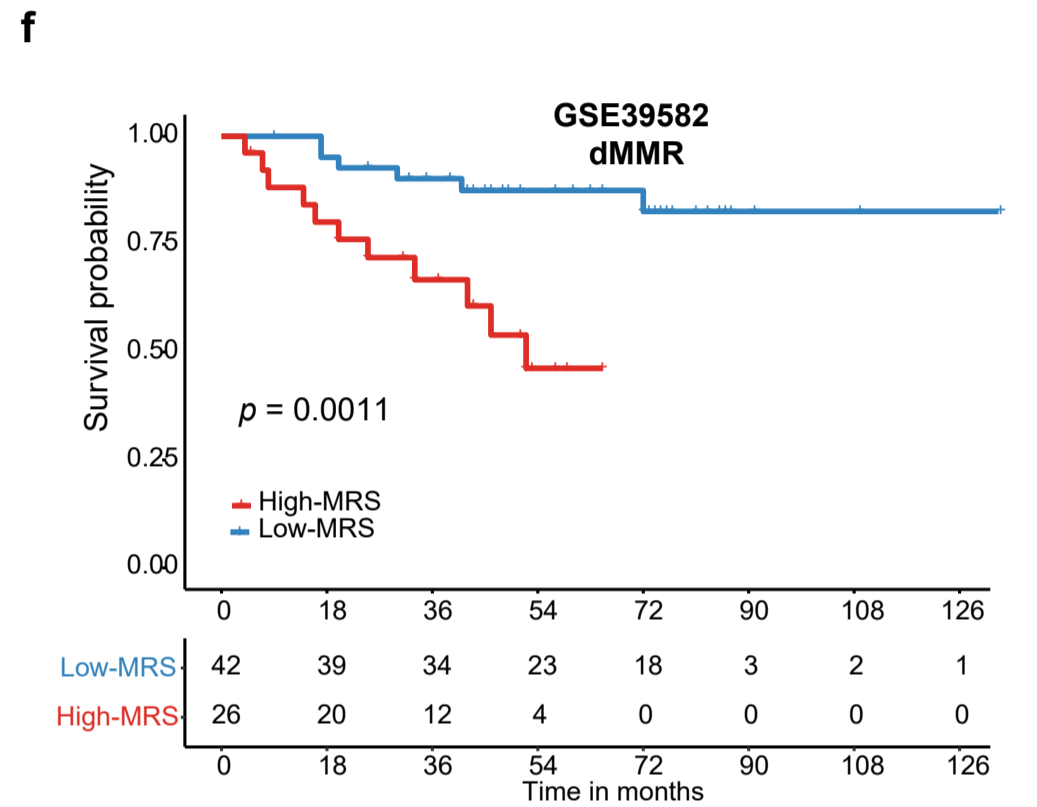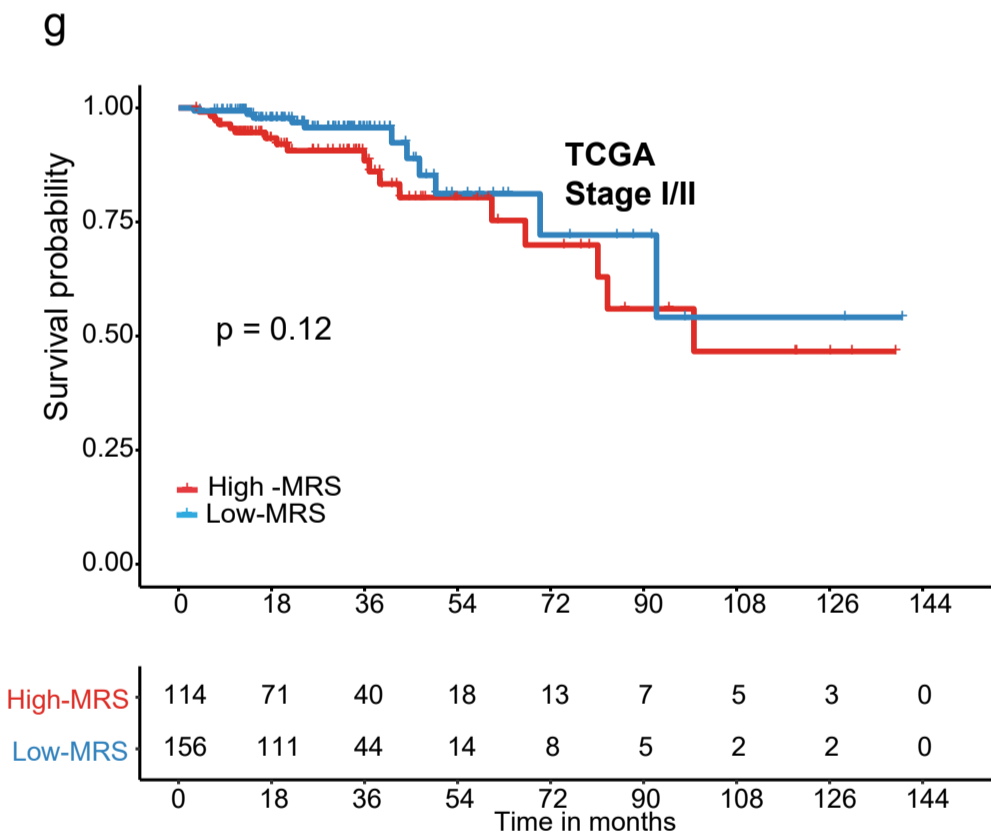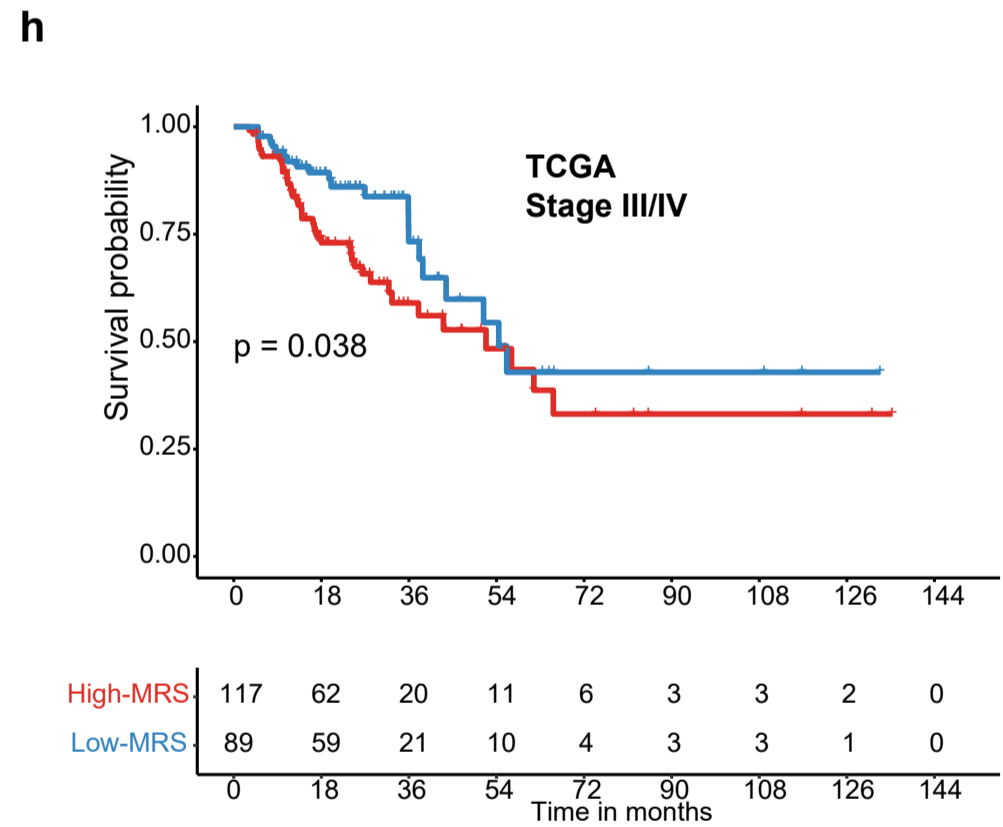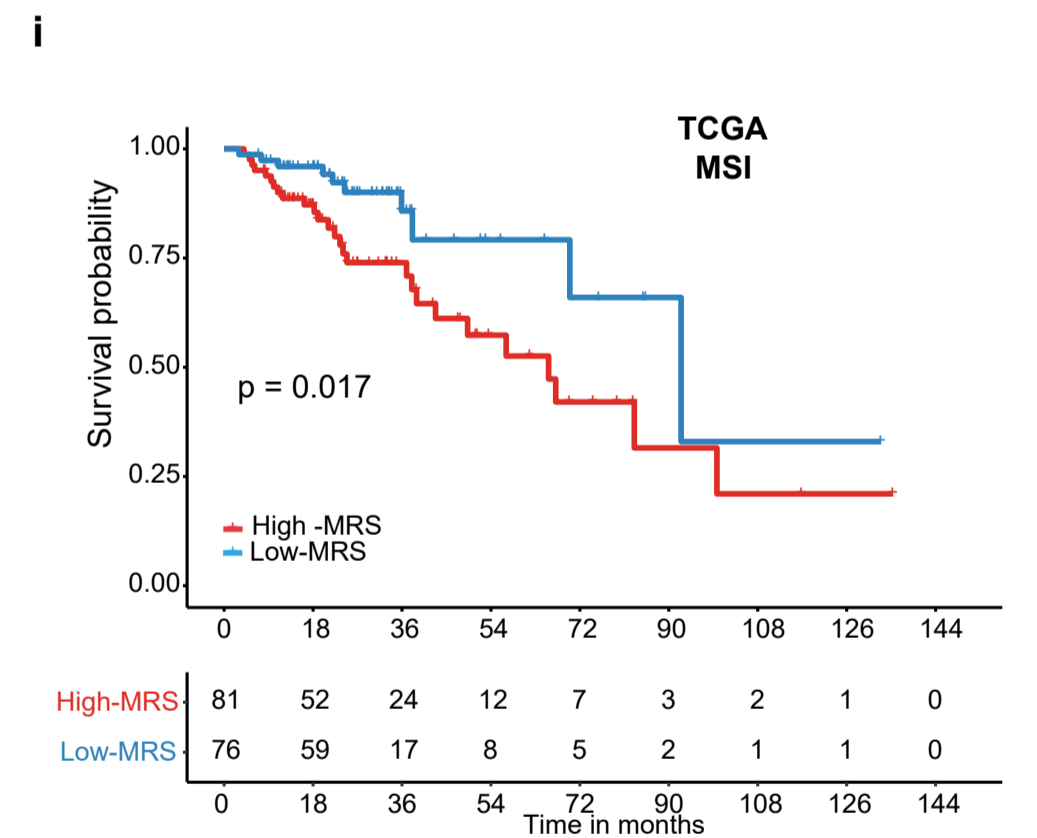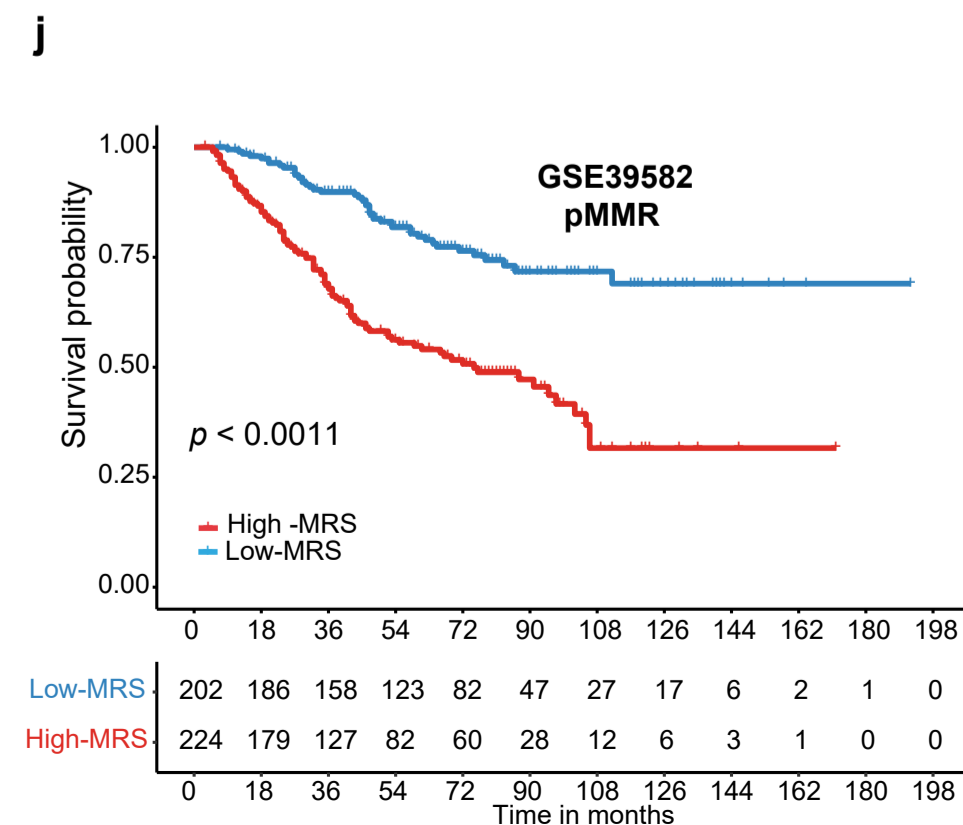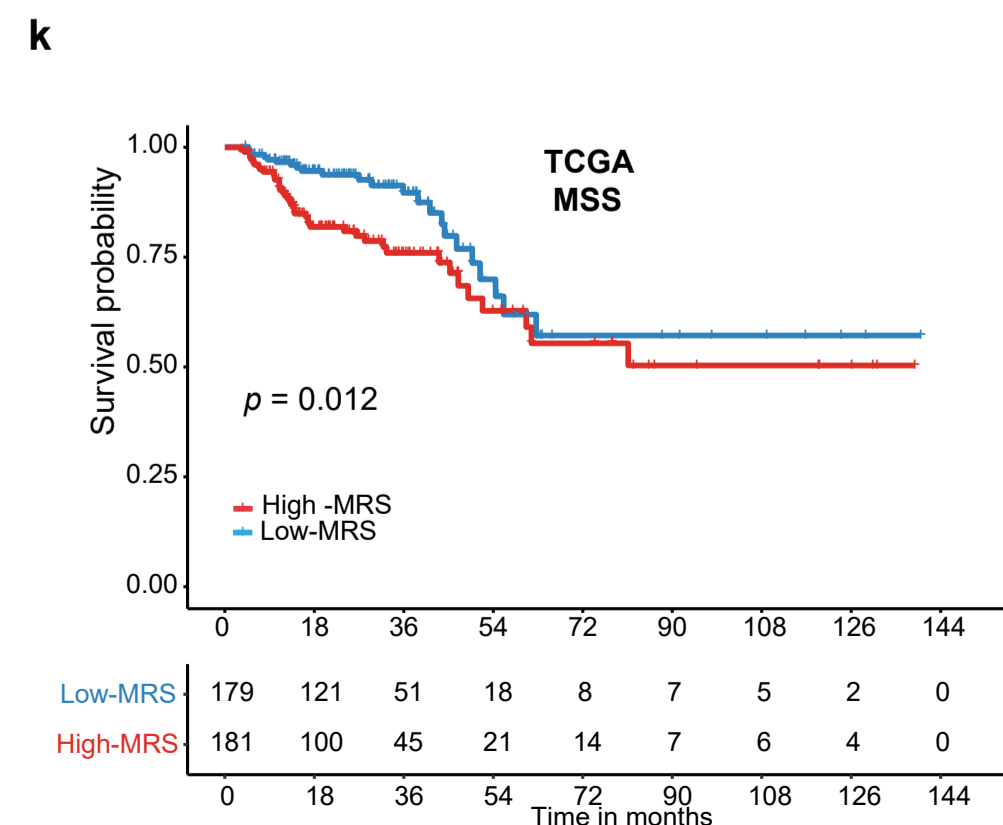

Supplement: Supplementary file 10 — Additional file 10: Figure S3. LASSO model and subgroup analysis of OS between the high- and low-MRS group in the GSE39582 and TCGA datasets. [file 12967_2021_2952_MOESM10_ESM.pdf]

**a**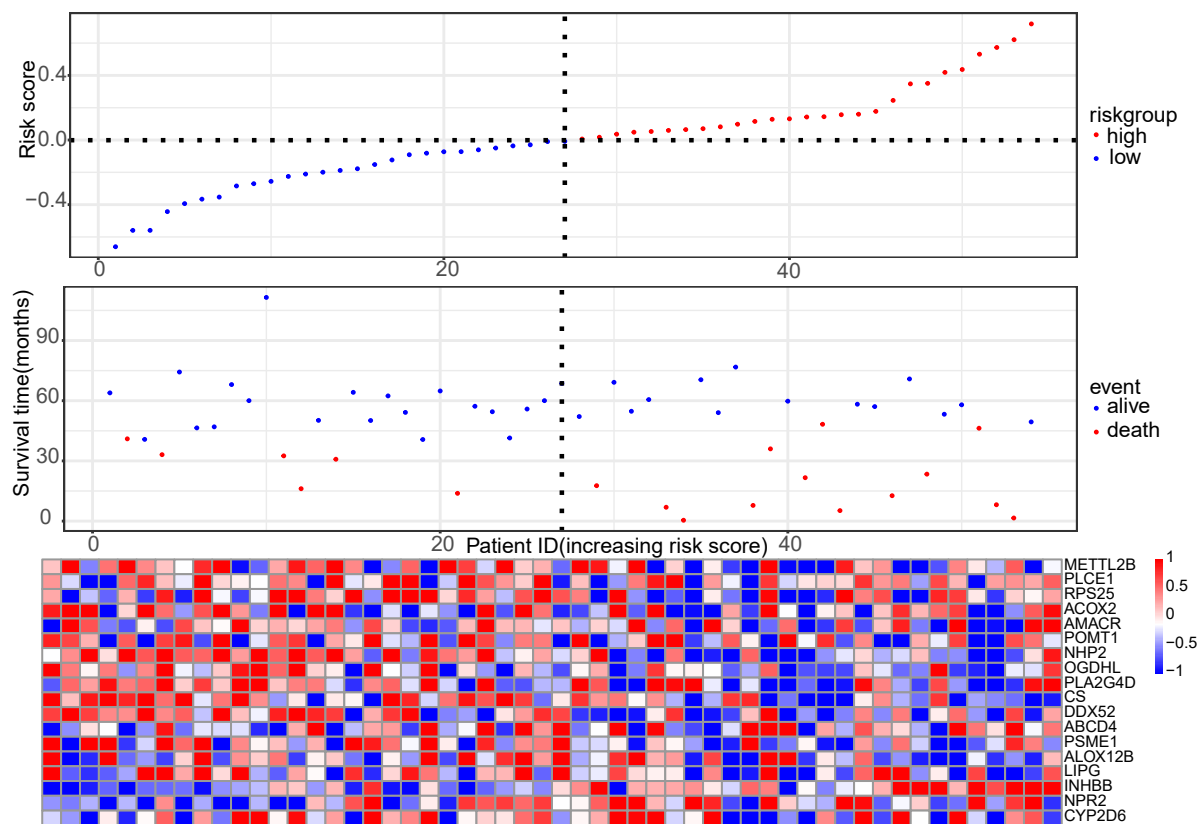**b**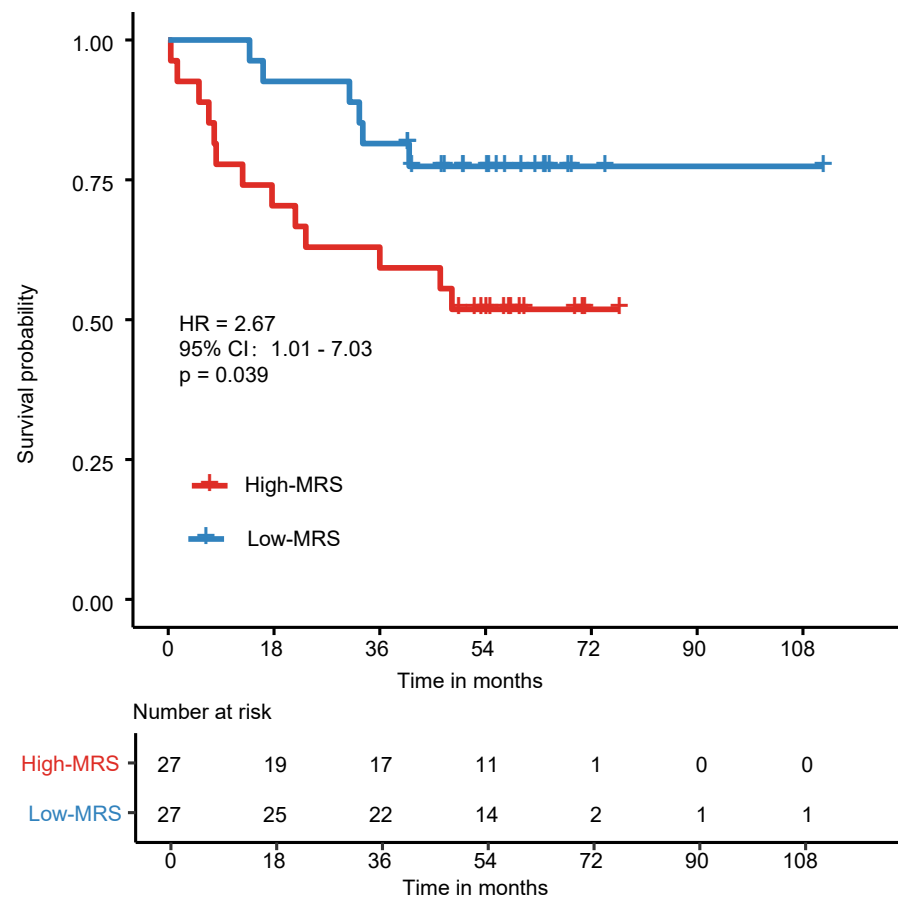**c**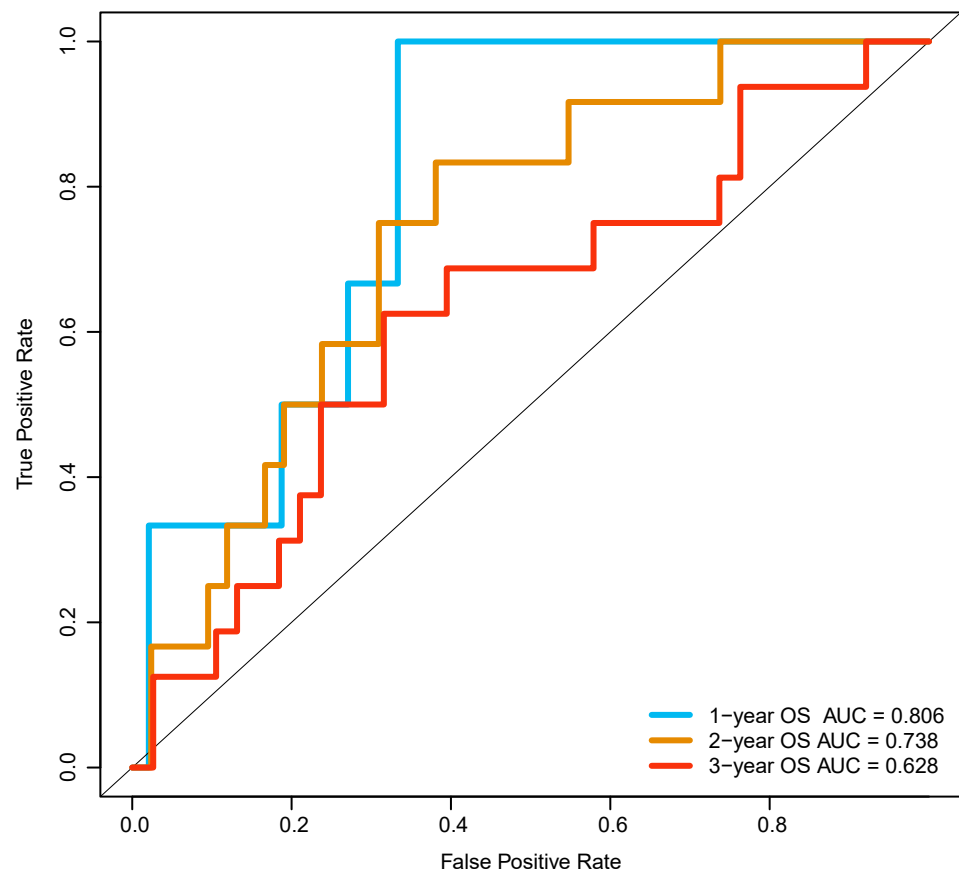

Supplement: Supplementary file 11 — Additional file 11: Figure S4. Validation of the MRS in the GSE17537 dataset. [file 12967_2021_2952_MOESM11_ESM.pdf]

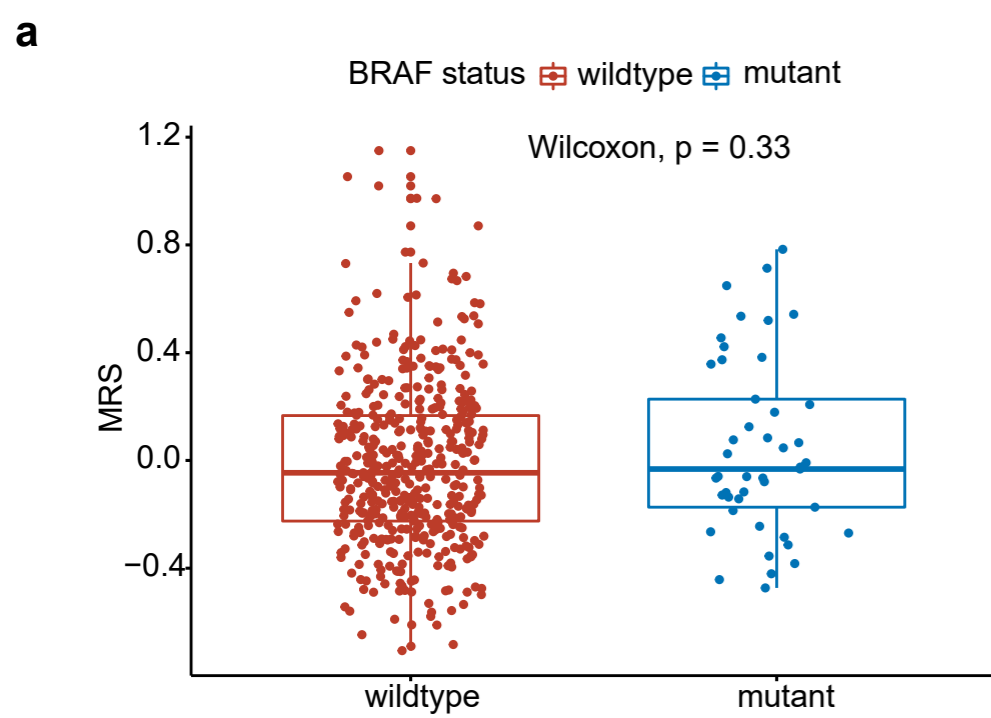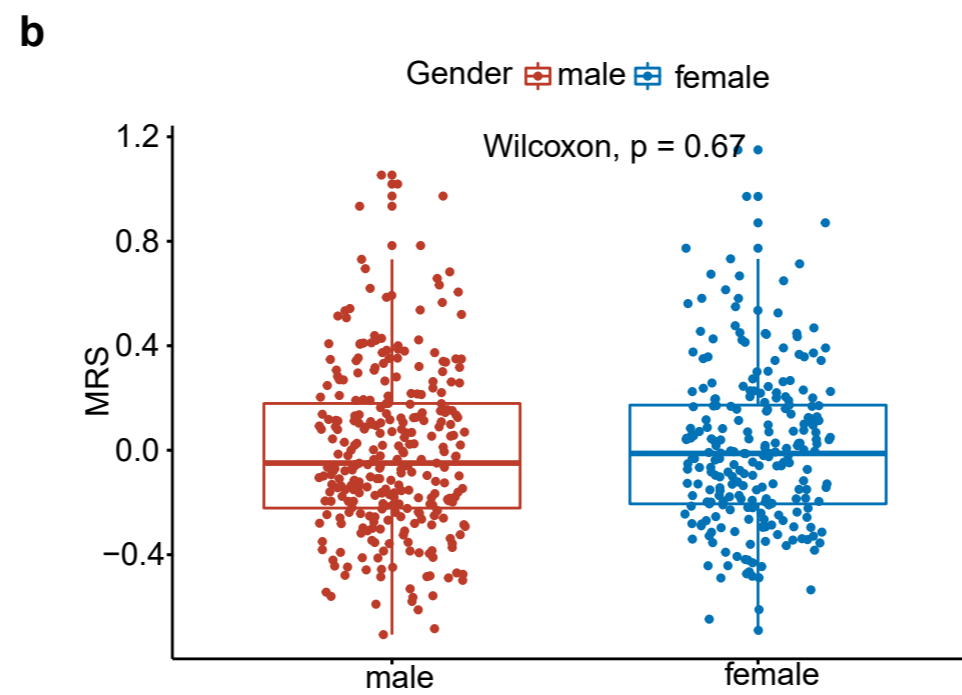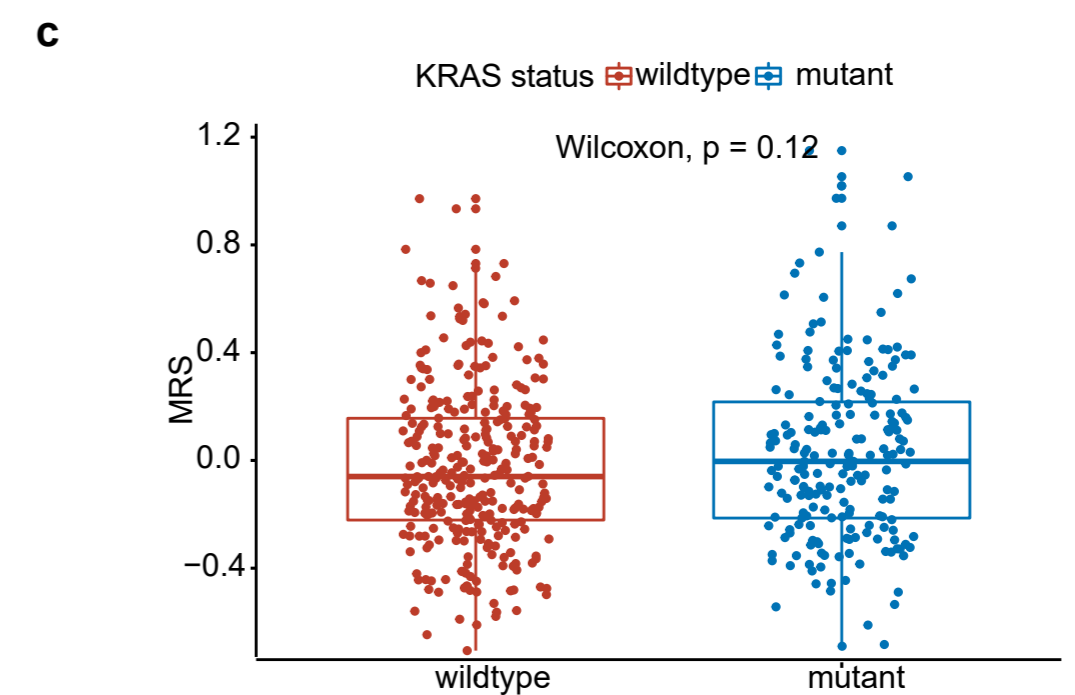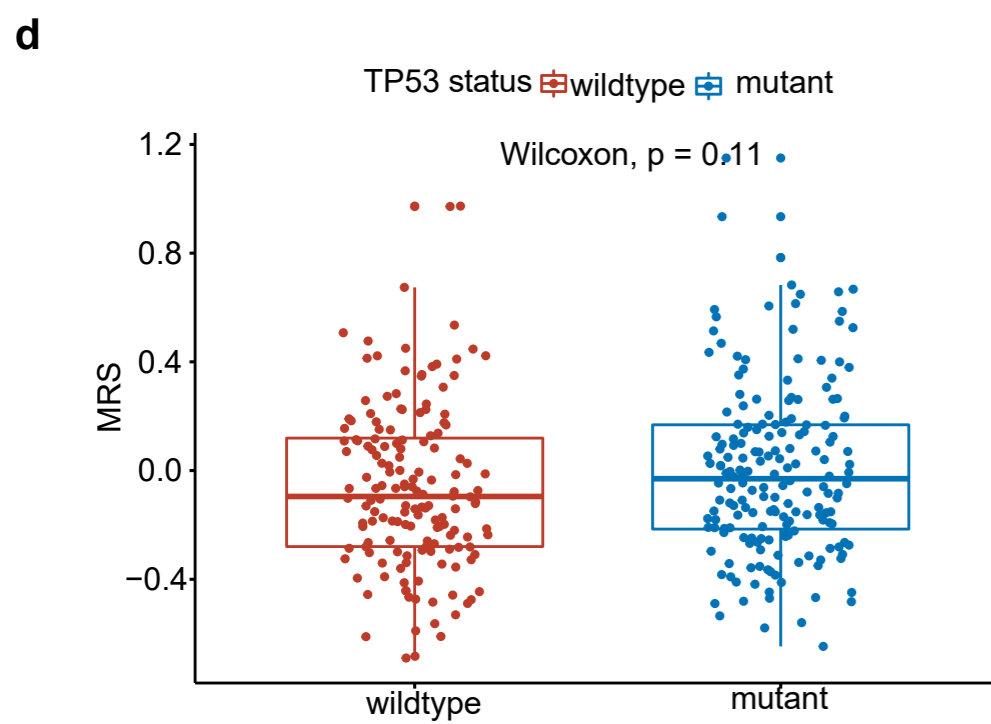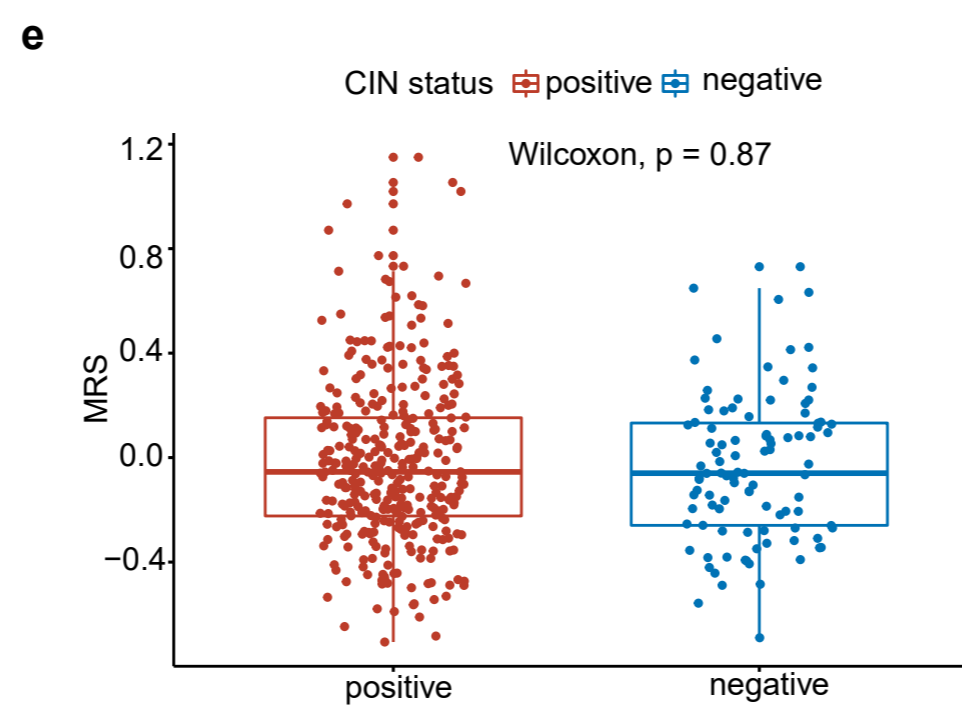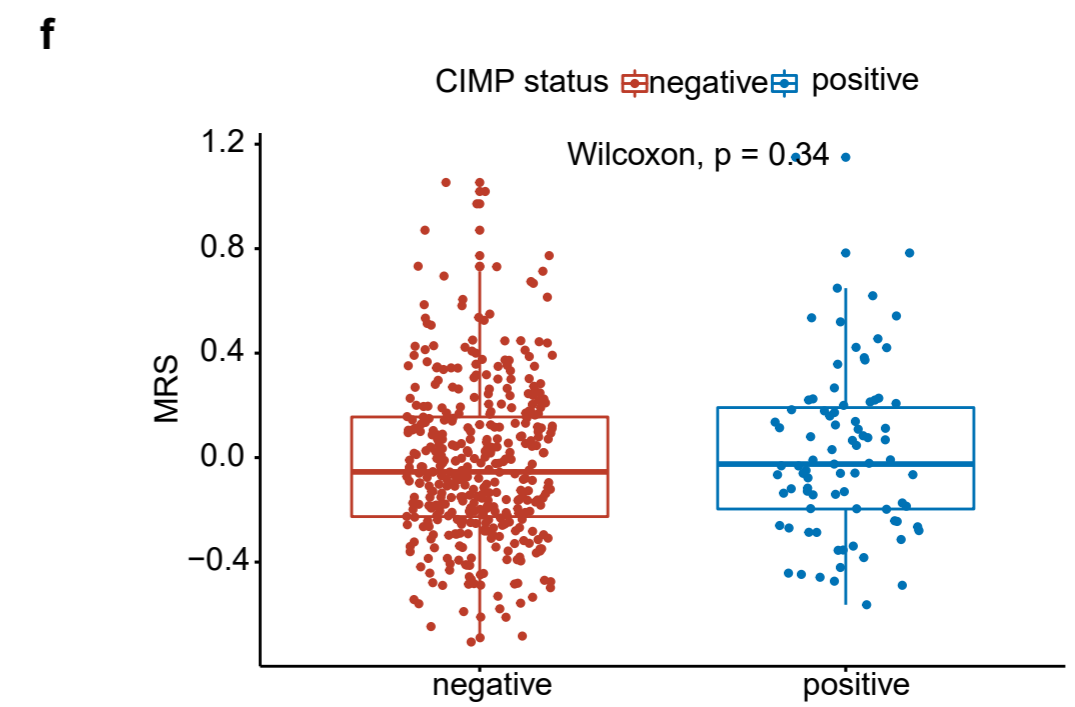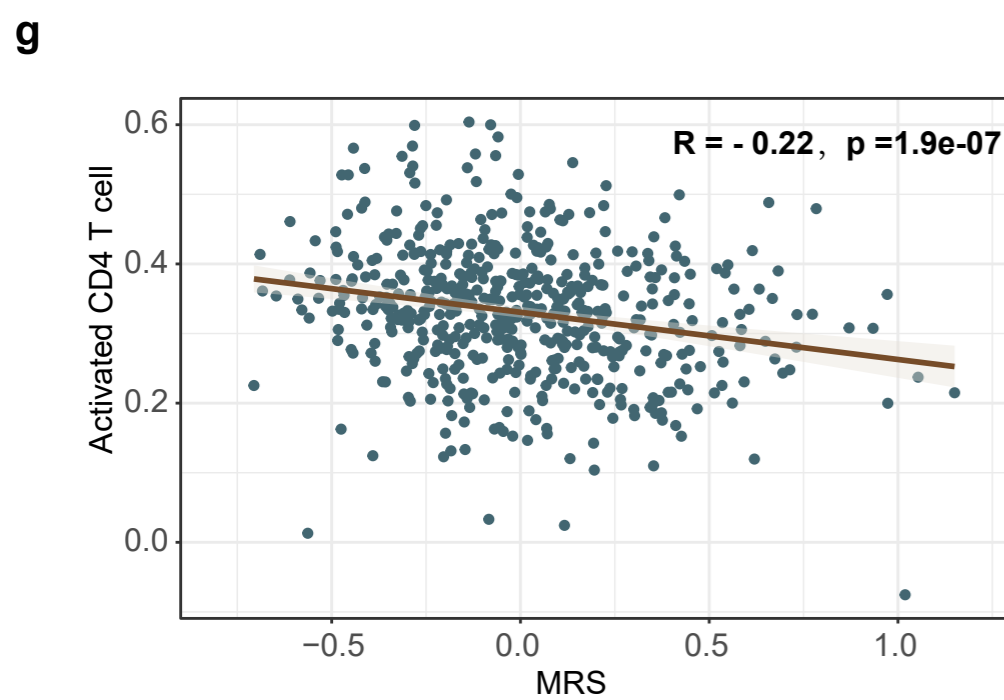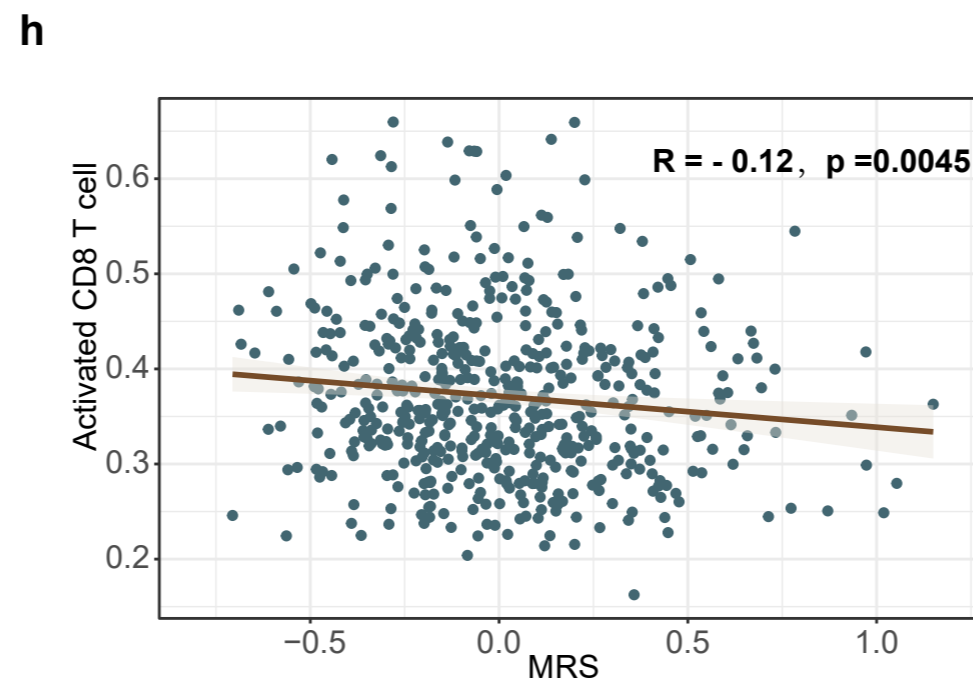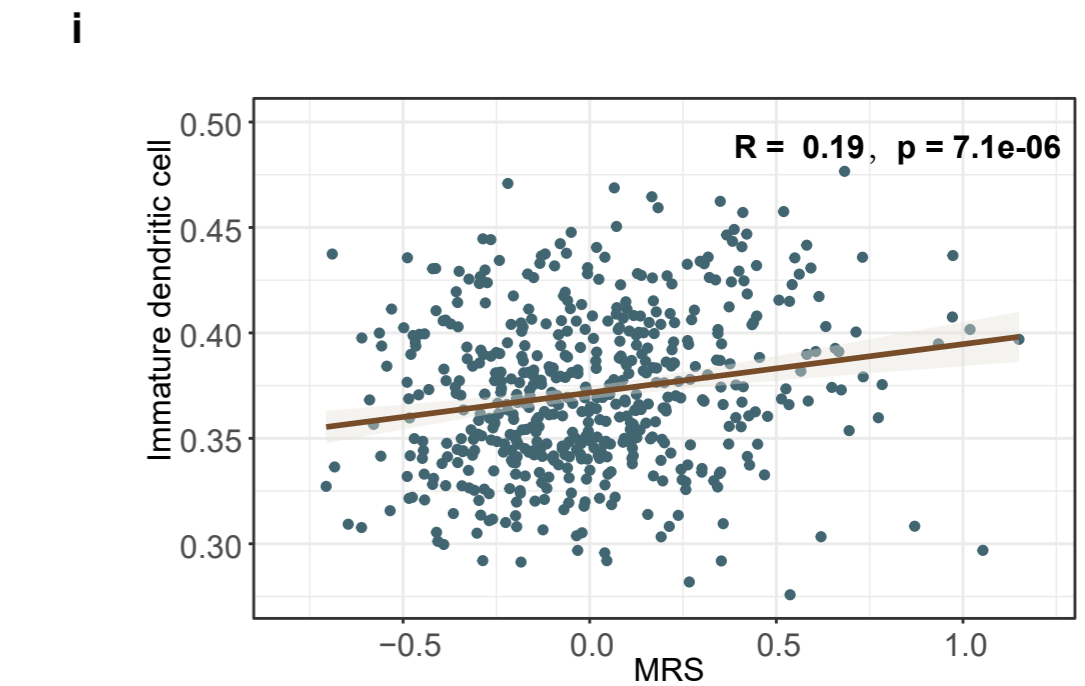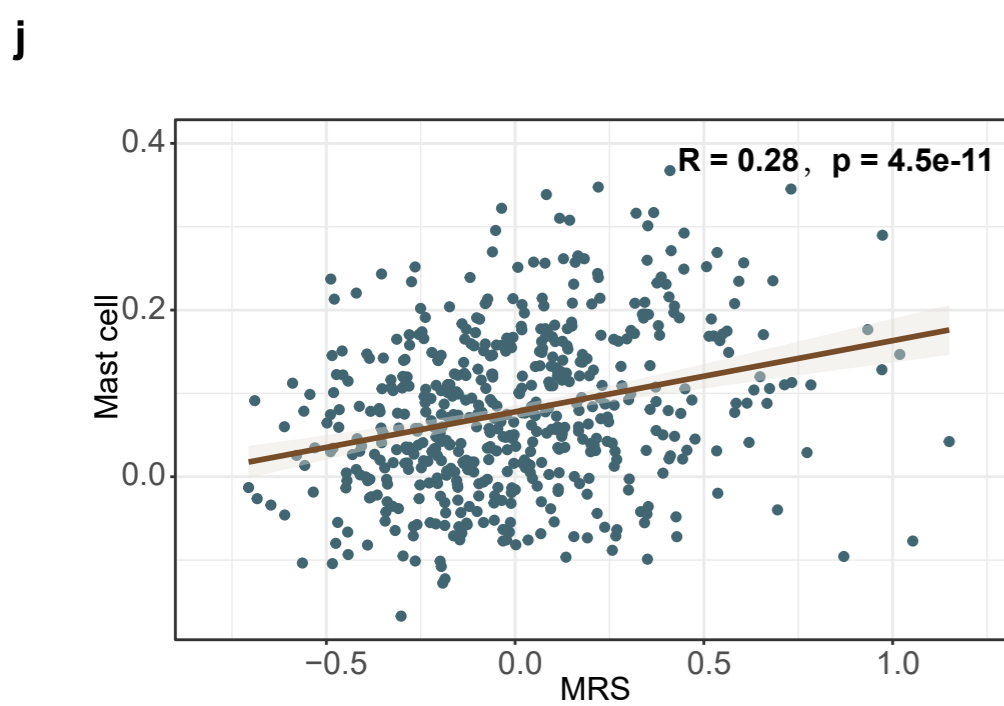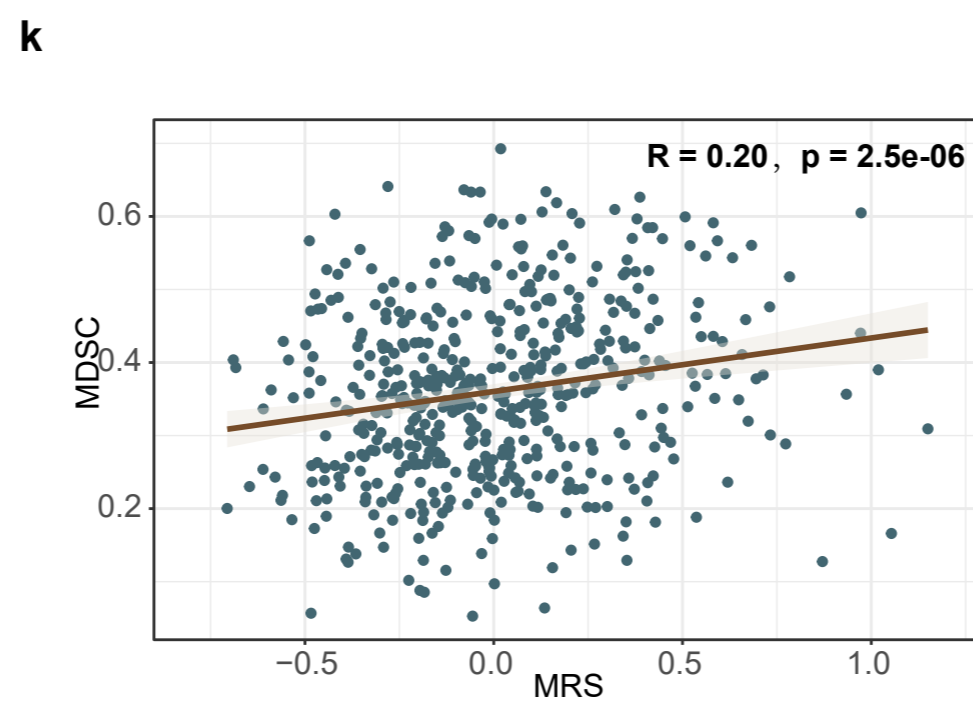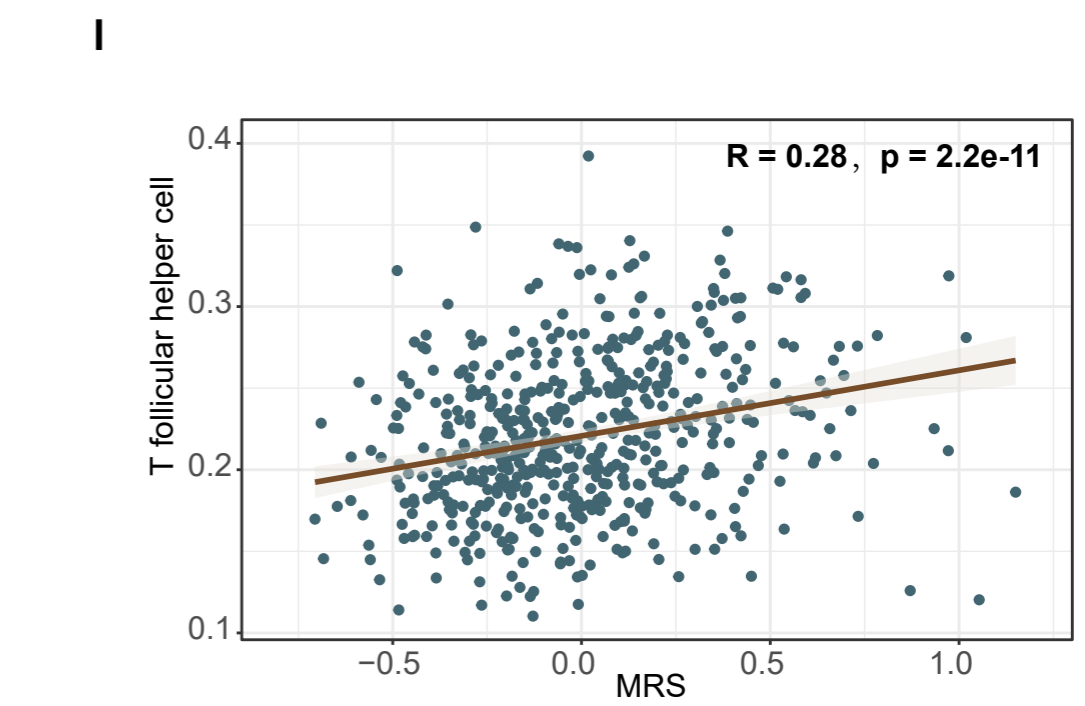

Supplement: Supplementary file 12 — Additional file 12: Figure S5. Spearman correlations between immune cells and the MRS and distribution of the MRS among various clinical variables. [file 12967_2021_2952_MOESM12_ESM.pdf]

**a**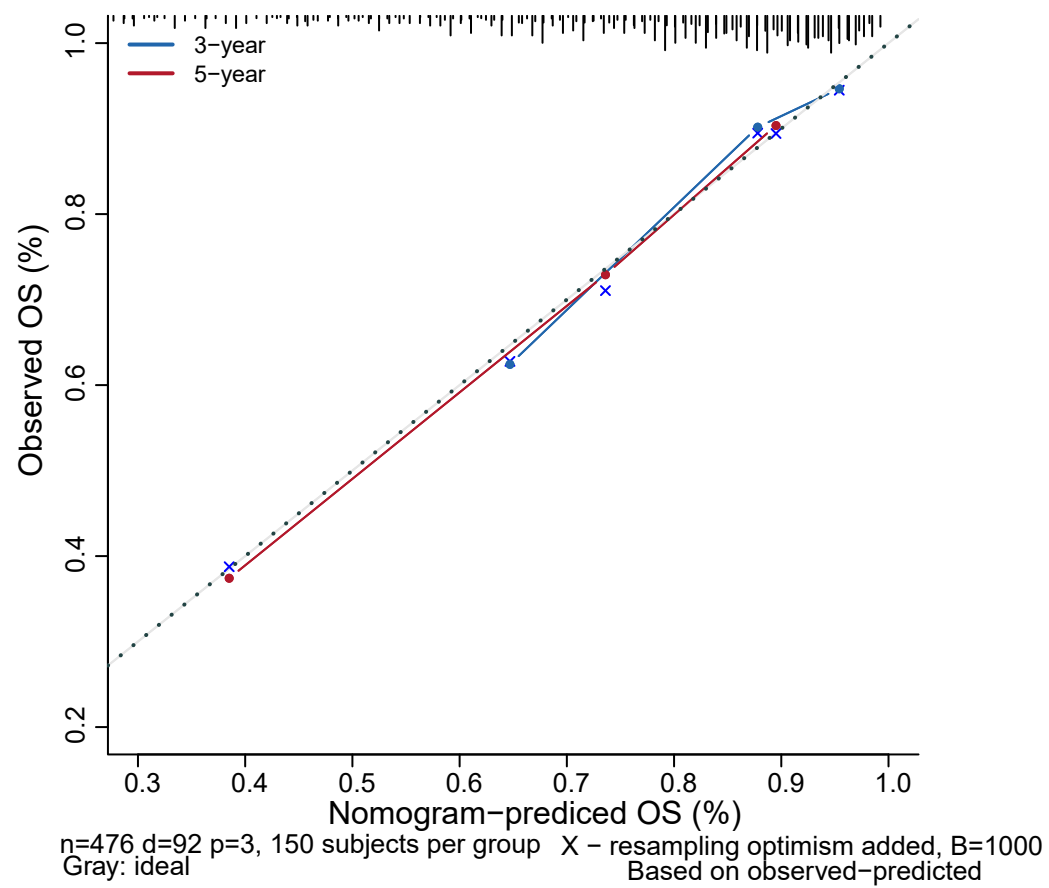**b**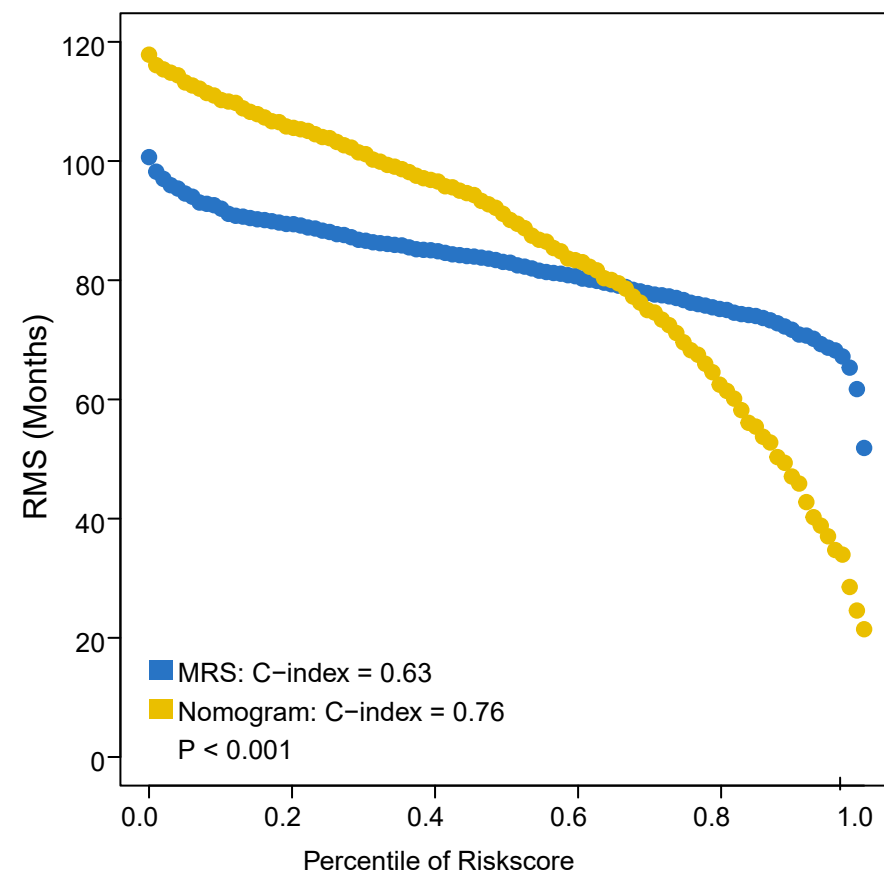**c**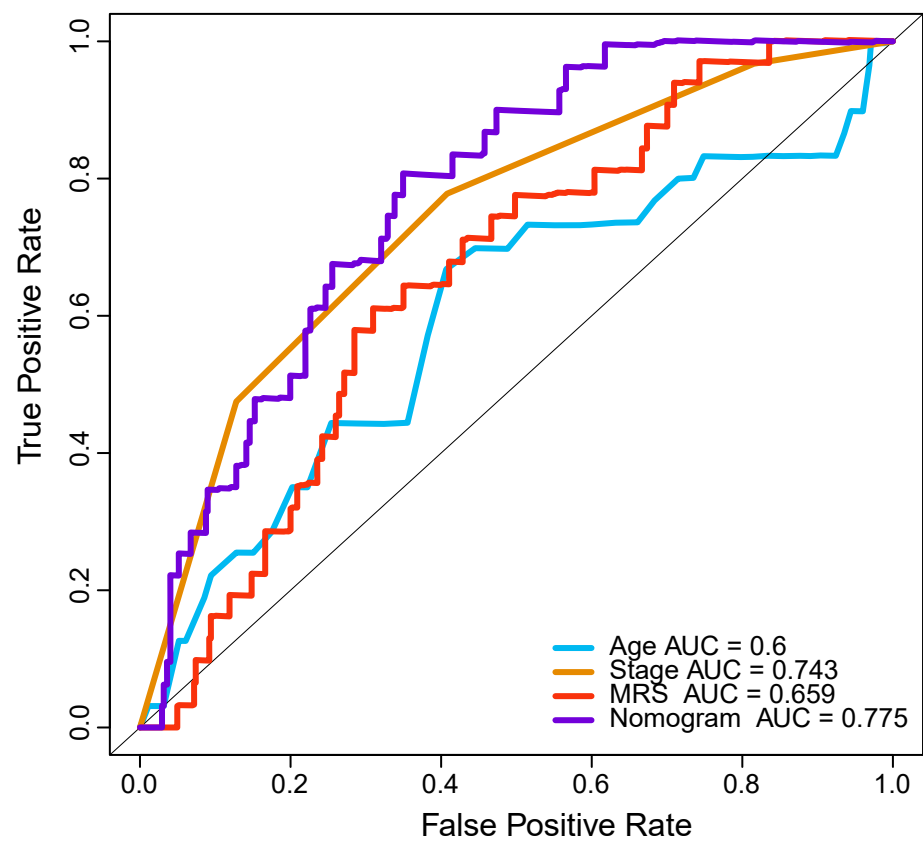**d**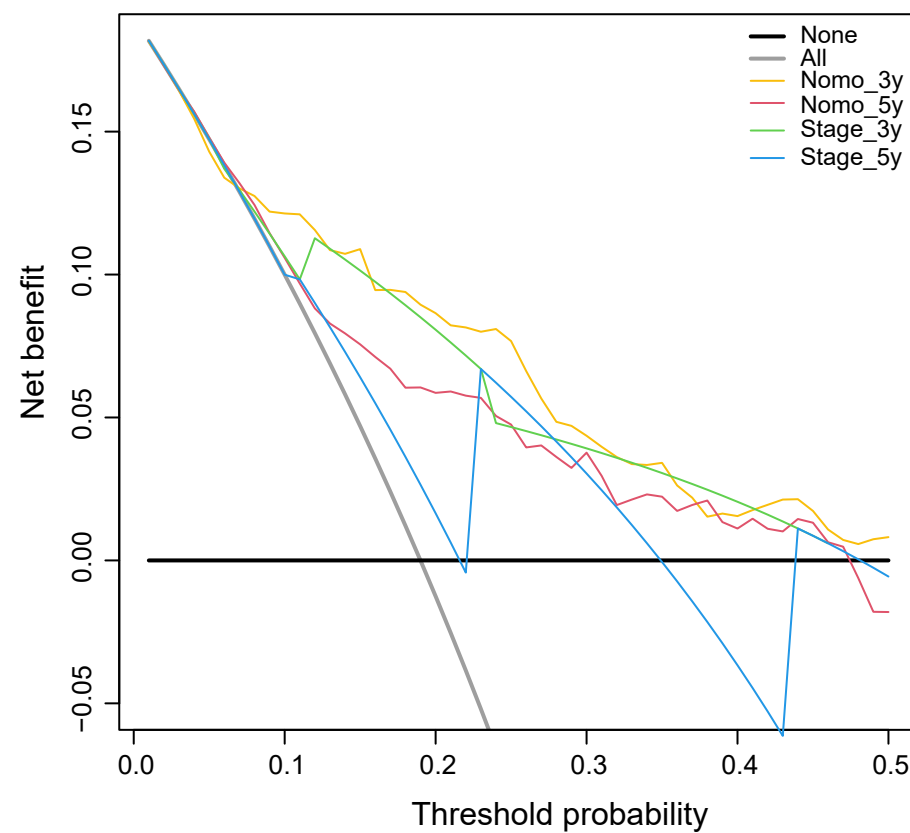

Supplement: Supplementary file 13 — Additional file 13: Figure S6. Validation of the nomogram in the TCGA CRC dataset. [file 12967_2021_2952_MOESM13_ESM.pdf]

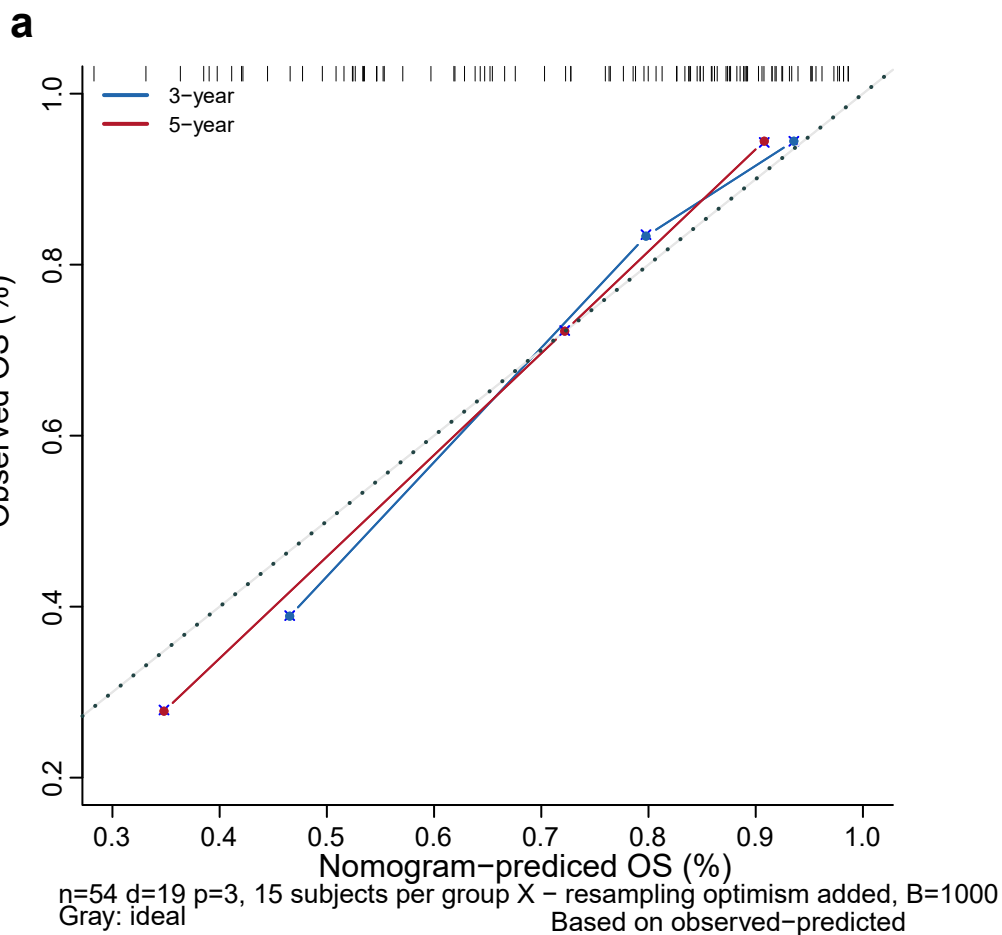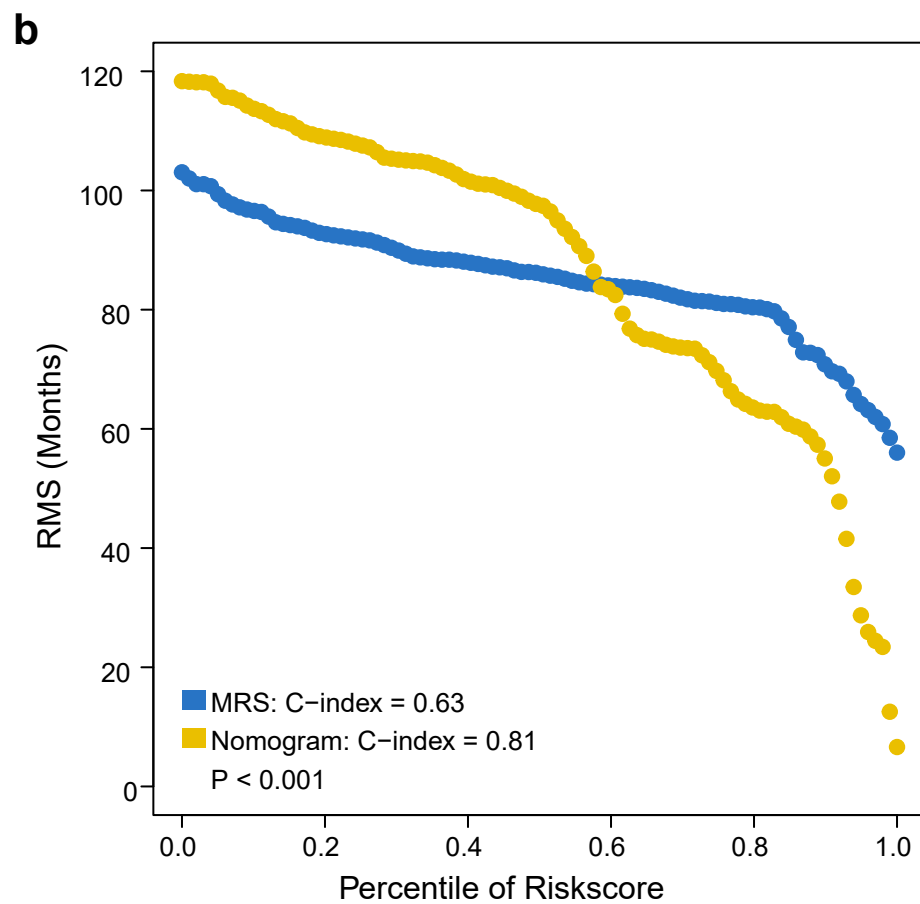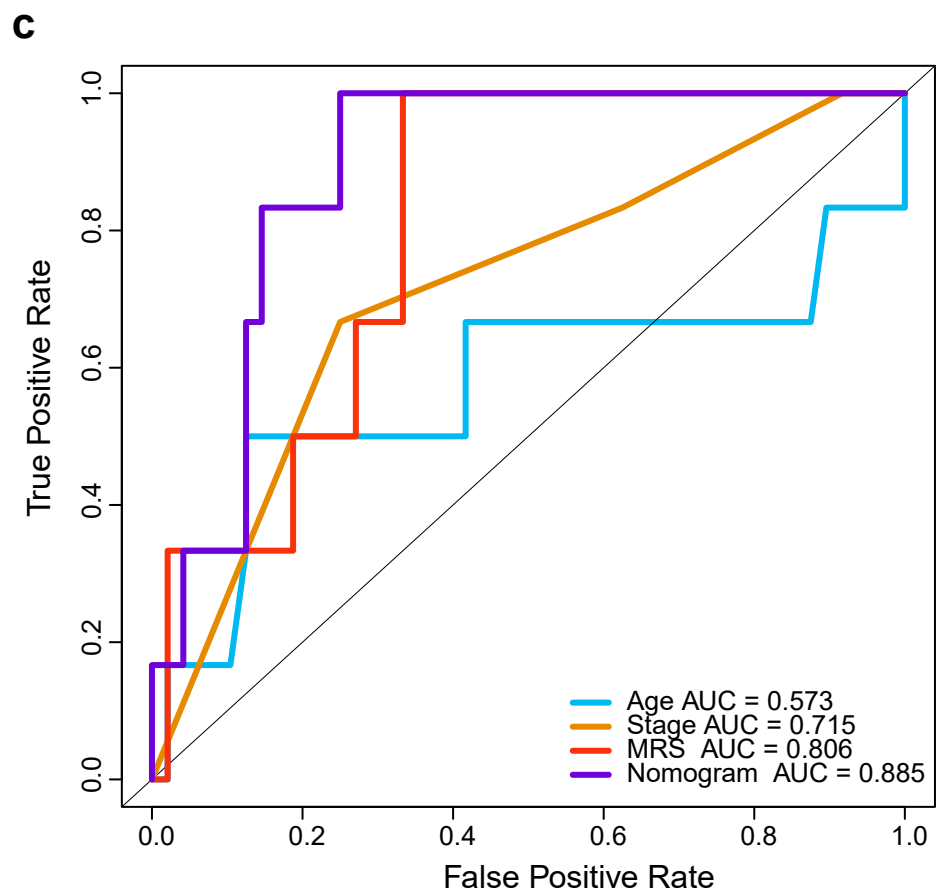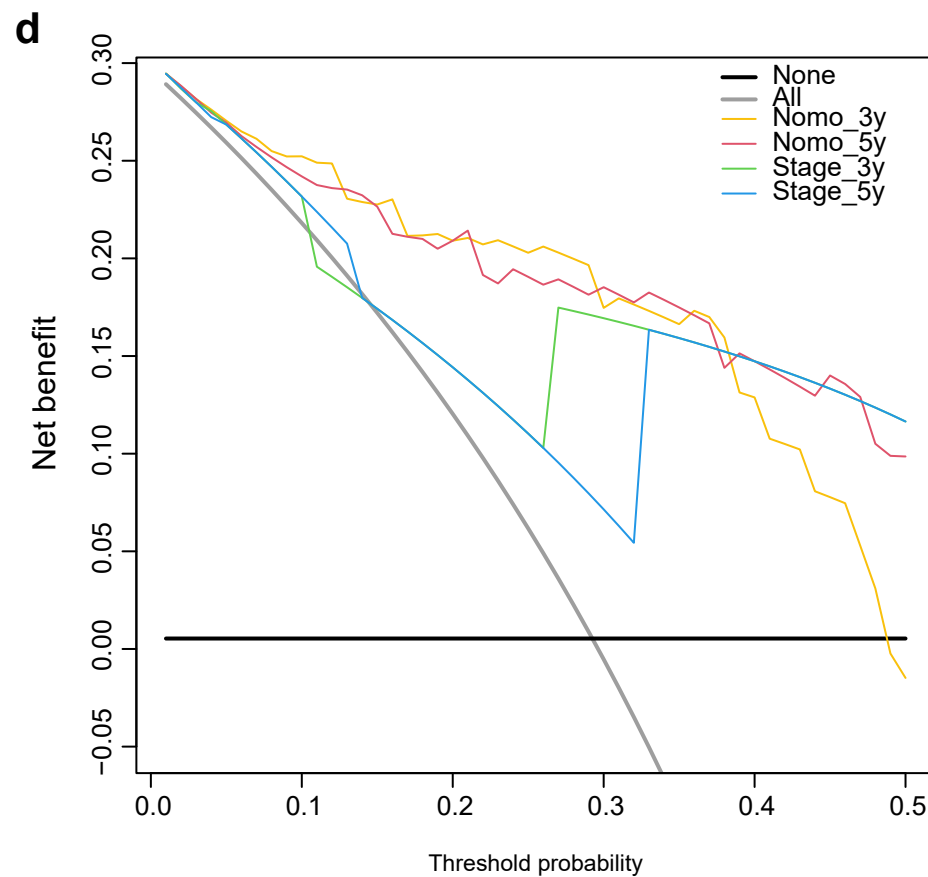

Supplement: Supplementary file 14 — Additional file 14: Figure S7. Validation of the nomogram in the GSE17537 dataset. [file 12967_2021_2952_MOESM14_ESM.pdf]

**a**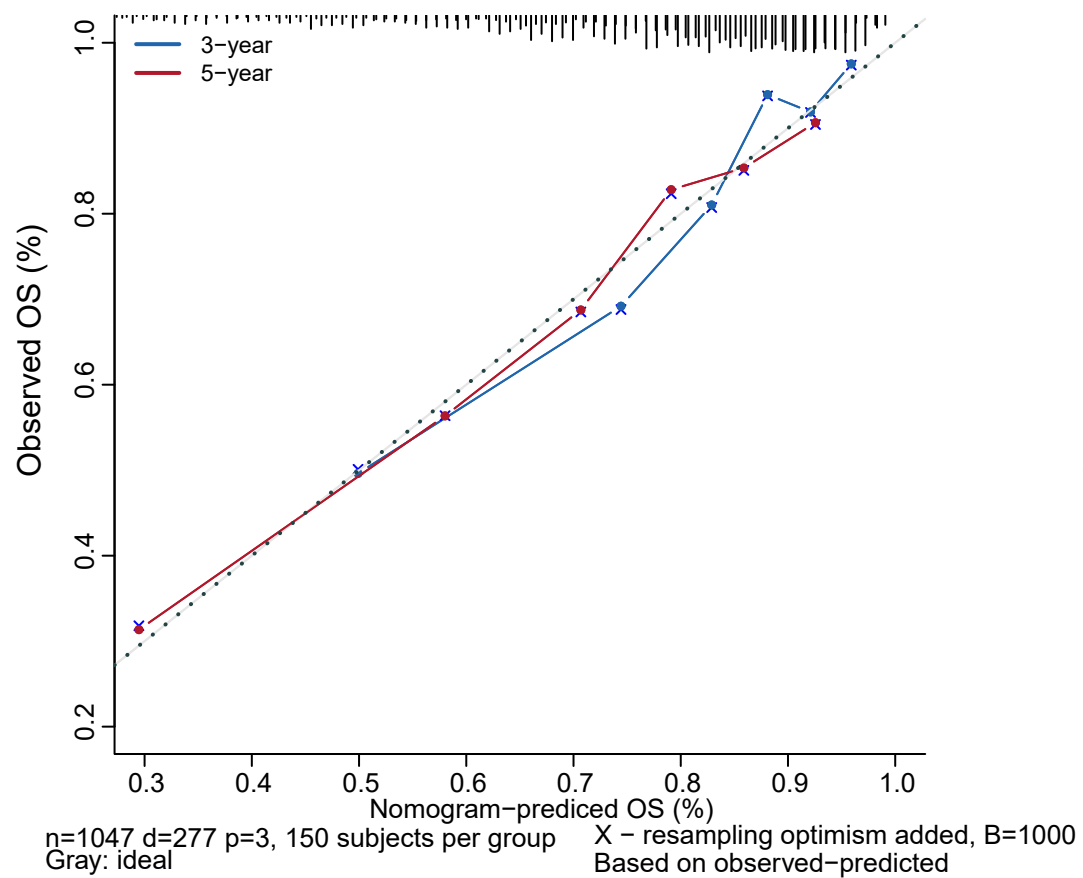**b**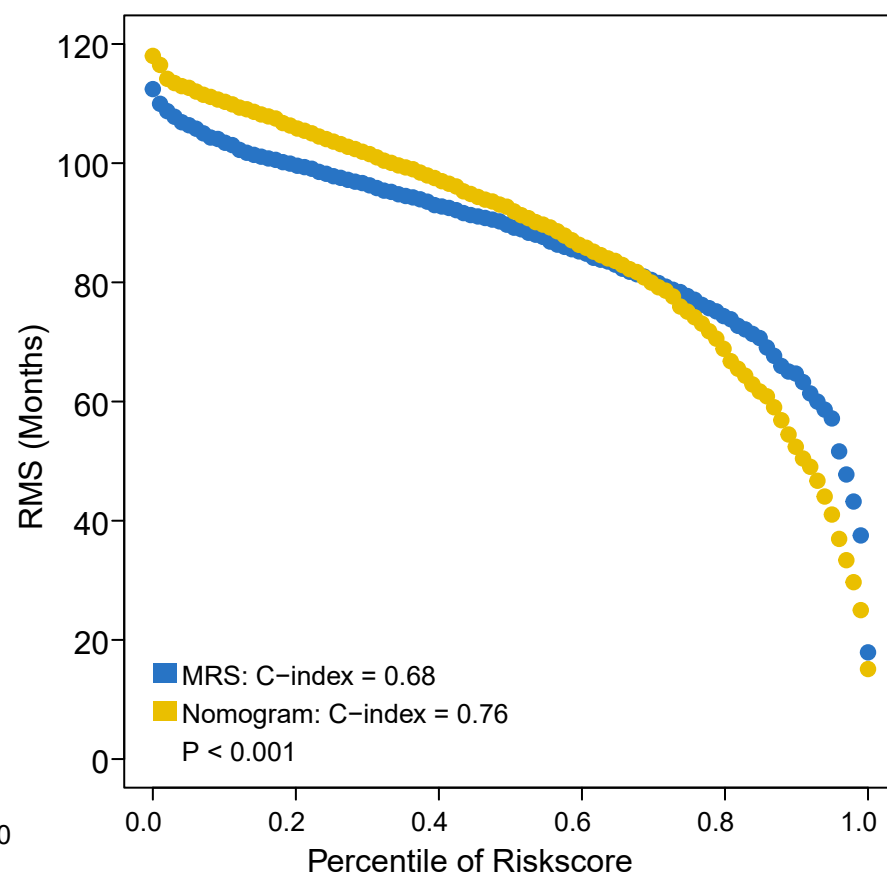**c**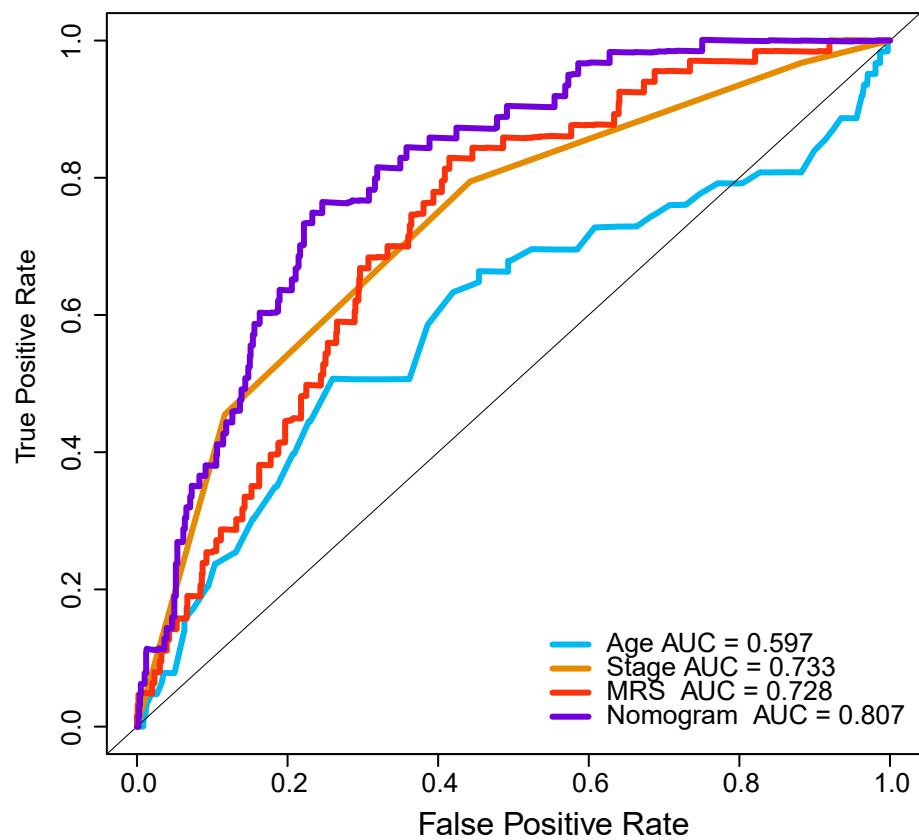**d**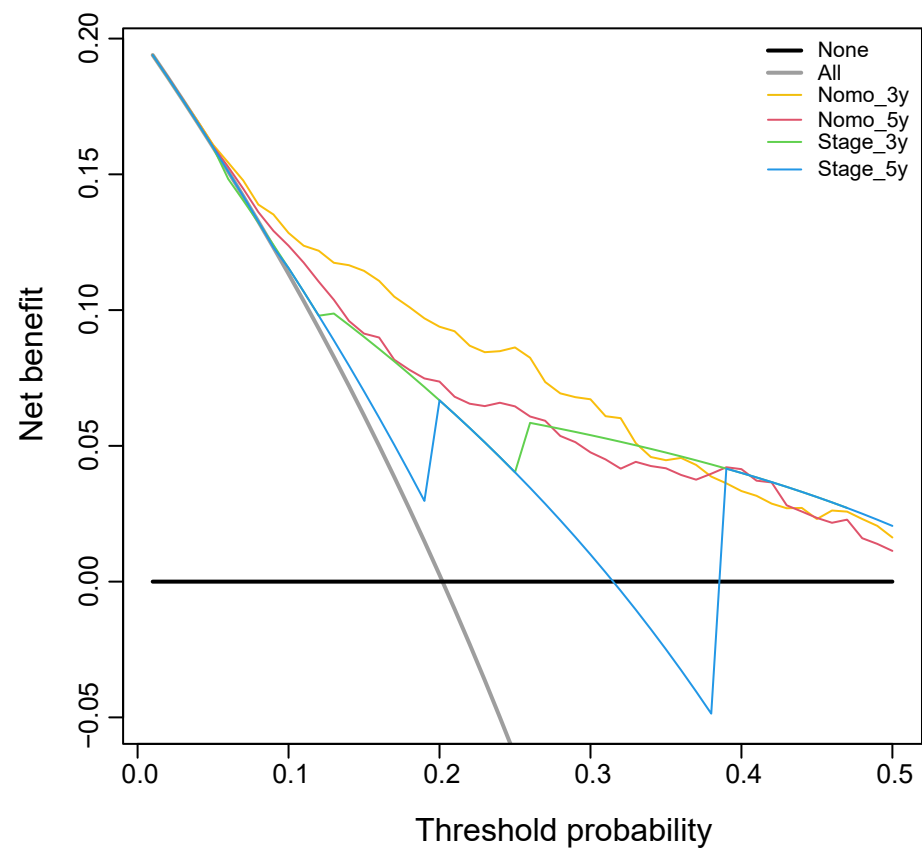

Supplement: Supplementary file 15 — Additional file 15: Figure S8. Validation of the nomogram in the entire dataset. [file 12967_2021_2952_MOESM15_ESM.pdf]
